# Epimural Indicator Phylotypes of Transiently-Induced Subacute Ruminal Acidosis in Dairy Cattle

**DOI:** 10.3389/fmicb.2016.00274

**Published:** 2016-03-04

**Authors:** Stefanie U. Wetzels, Evelyne Mann, Barbara U. Metzler-Zebeli, Poulad Pourazad, Muhammad Qumar, Fenja Klevenhusen, Beate Pinior, Martin Wagner, Qendrim Zebeli, Stephan Schmitz-Esser

**Affiliations:** ^1^Department for Farm Animals and Veterinary Public Health, Institute of Animal Nutrition and Functional Plant Compounds, University of Veterinary Medicine ViennaVienna, Austria; ^2^Department of Farm Animal and Public Health in Veterinary Medicine, Institute for Milk Hygiene, Milk Technology and Food Science, University of Veterinary Medicine ViennaVienna, Austria; ^3^Department for Farm Animals and Veterinary Public Health, Research Cluster Animal Gut Health, University of Veterinary Medicine ViennaVienna, Austria; ^4^Department for Farm Animals and Veterinary Public Health, University Clinic for Swine, University of Veterinary Medicine ViennaVienna, Austria; ^5^Department for Farm Animals and Veterinary Public Health, Institute for Veterinary Public Health, University of Veterinary Medicine ViennaVienna, Austria; ^6^Department of Animal Science, Iowa State UniversityAmes, IA, USA

**Keywords:** *Campylobacter*, *Kingella*, rumen epithelium, microbiota, long-term subacute rumen acidosis, cattle, feeding, 16S rRNA gene amplicon sequencing

## Abstract

The impact of a long-term subacute rumen acidosis (SARA) on the bovine epimural bacterial microbiome (BEBM) and its consequences for rumen health is poorly understood. This study aimed to investigate shifts in the BEBM during a long-term transient SARA model consisting of two concentrate-diet-induced SARA challenges separated by a 1-week challenge break. Eight cows were fed forage and varying concentrate amounts throughout the experiment. In total, 32 rumen papilla biopsies were taken for DNA isolation (4 sampling time points per cow: at the baseline before concentrate was fed, after the first SARA challenge, after the challenge break, and after the second SARA challenge). Ruminal pH was continuously monitored. The microbiome was determined using Illumina MiSeq sequencing of the 16S rRNA gene (V345 region). In total 1,215,618 sequences were obtained and clustered into 6833 operational taxonomic units (OTUs). *Campylobacter* and *Kingella* were the most abundant OTUs (16.5 and 7.1%). According to ruminal pH dynamics, the second challenge was more severe than the first challenge. Species diversity estimates and evenness increased during the challenge break compared to all other sampling time points (*P* < 0.05). During both SARA challenges, *Kingella-* and *Azoarcus*-OTUs decreased (0.5 and 0.4 fold-change) and a dominant *Ruminobacter*-OTU increased during the challenge break (18.9 fold-change; *P* < 0.05). qPCR confirmed SARA-related shifts. During the challenge break noticeably more OTUs increased compared to other sampling time points. Our results show that the BEBM re-establishes the baseline conditions slower after a SARA challenge than ruminal pH. Key phylotypes that were reduced during both challenges may help to establish a bacterial fingerprint to facilitate understanding effects of SARA conditions on the BEBM and their consequences for the ruminant host.

## Introduction

The bovine epimural bacterial microbiome (BEBM) in the rumen exerts a multitude of physiologically important functions (Cheng and Wallace, 1979; Dinsdale et al., 1980; McCowan et al., 1980). This bacterial community is at the interface between rumen content and the host and is distinct from the bacterial community in rumen fluid and mat (Cheng and McAllister, 1997). Epimural bacteria can compete with adhesive, putative pathogenic microorganisms and can provide protection against harmful microbes, e.g., by forming a protective biofilm on the ruminal mucosa (Hungate, 1966; Kamra, 2005). Previous data suggest *Atopobium, Desulfocurvus, Fervidicola, Eubacterium, Lactobacillus, Micrococcus, Staphylococcus, Streptococcus, Corynebacterium*, and *Propionibacterium* to be highly abundant in the BEBM in the rumen (Cheng and Wallace, 1979; Petri et al., 2013). In general, comparatively little is known about the influence of diet on the BEBM and its role in the development and prevention of metabolic disorders.

One common severe metabolic disorder of cattle is subacute rumen acidosis (SARA) which has been described as a condition with intermittent phases of ruminal pH below 5.8 for more than 300 min daily (Zebeli et al., 2008). SARA is caused when cattle are fed with a high grain diet to support rapid growth rates or high milk production (Zebeli et al., 2012; Boerman et al., 2015). This disorder is associated with major microbial and physiological changes in the rumen that increase the risk of systemic metabolic disorders in cattle (Zebeli and Metzler-Zebeli, 2012), causing significant economic losses in the cattle industry (Plaizier et al., 2008).

So far, the BEBM has been investigated during SARA challenges when cattle were fed high-grain diets during 1 or 2 weeks without interruption (Chen et al., 2011; Petri et al., 2013). This type of feeding implies continuous SARA conditions and can be referred to as persistent SARA. Because cows were not allowed to recover between acidotic insults of the rumen in these studies, it is not clear whether the results are representative for the typical, chronically intermittent course of the metabolic disorder. In dairy herds, cows usually experience interrupted episodes of SARA as they attempt to select roughage feed during SARA to counteract the drop in ruminal pH (Hendriksen et al., 2015; Humer et al., 2015). The influence of a long-term SARA challenge on the ruminal epimural microbiome using a transient feeding model was not investigated until now. In-depth sequencing already helped identifying key phylotypes for several metabolic disorders in humans and animals, thereby improving our understanding of the etiopathogenesis of different disorders (Liang et al., 2014). Therefore, identification of key taxa might lead to a better understanding and development of prevention strategies for metabolic disorders like SARA in cattle (Petri et al., 2013; Ze et al., 2013; Wetzels et al., 2015). For this reason we conducted a long-term experiment with rumen-cannulated cows using a transient SARA challenge model which was characterized by two SARA challenges which were separated by a challenge break of 1 week. The objective of the present study was to monitor the shifts in BEBM taxa during the first and second periods of SARA. The underlying hypothesis of our study was that the BEBM shows distinct shifts in the community composition during dietary adaptation from forage-based to concentrate-based diet and that these shifts are less pronounced in the second and longer SARA challenge because of an adaptation that might occur after the first challenge and the following 1-week challenge break.

## Materials and methods

### Ethics statement

The animal experimental protocol of this research was discussed and approved by the institutional ethics committee of the University of Veterinary Medicine Vienna in accordance with Good Scientific Practice guidelines and the national authority according to ^§^26 of Law for Animal Experiments, Tierversuchsgesetz—TVG 2012 (GZ 68.205/0093-II/3b/2013).

### Animals, diets, and experimental design

**Figure 1:**
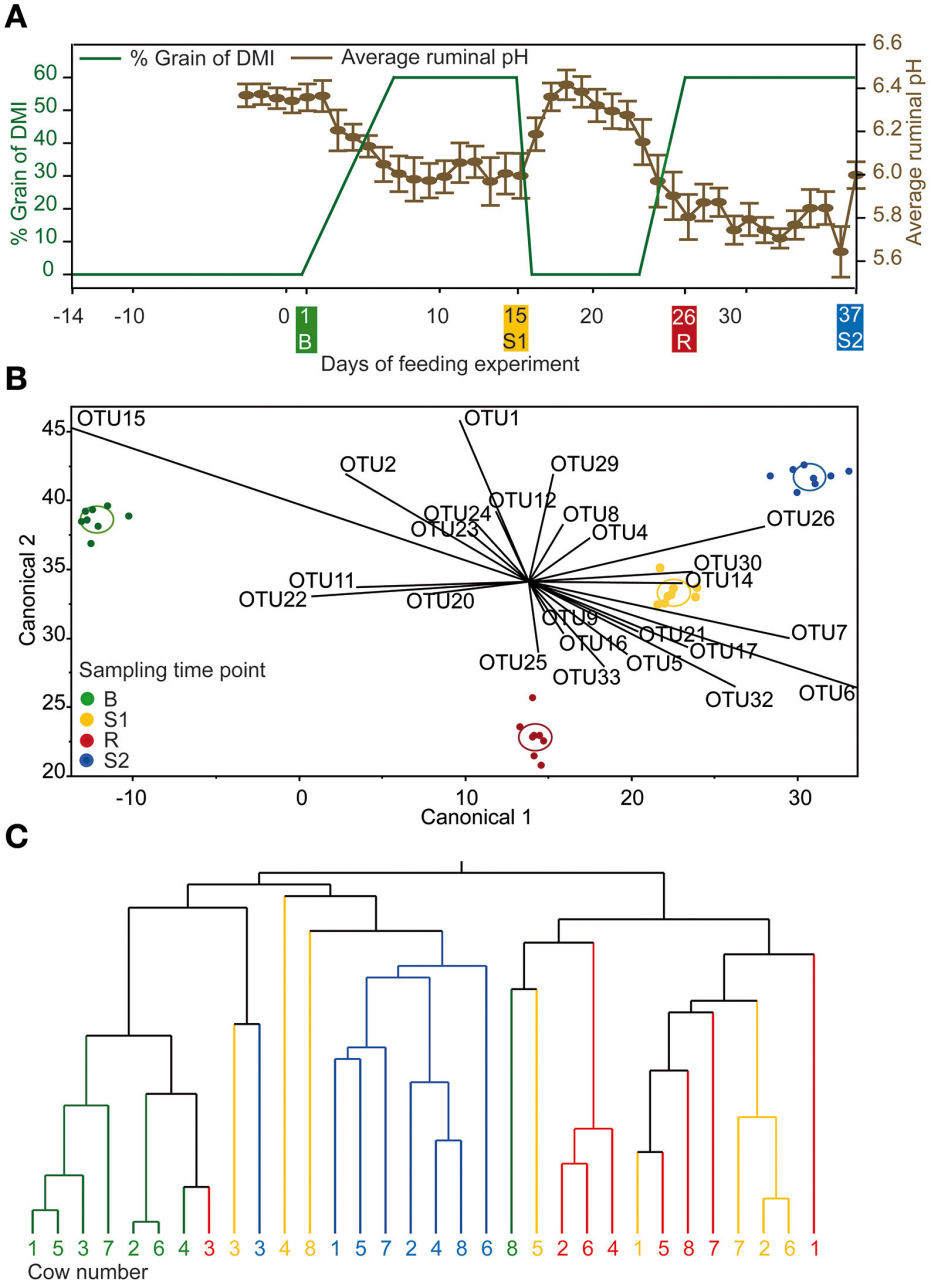
**(A)** Experimental design for the feeding experiment illustrated in green and daily mean ruminal pH illustrated in brown (±standard error). Sampling time points B (baseline), S1 (SARA 1), CB (challenge break), and S2 (SARA 2) are marked in same color in all panels. **(B)** Linear discriminant analysis with OTUs detected in every sampling time point displayed as canonical diagram (*n* = 270). **(C)** Hierarchic dendrogram with OTUs detected in every sampling time point (*n* = 270; method = ward).

In this long-term feeding experiment, eight rumen-cannulated (100 mm inner diameter Bar Diamond) non-lactating Holstein cows [initial body weight (BW): 710 ± 118 kg, mean ± SD] were used. Animals were kept together in a loose housing barn with straw bedding at the research farm of the University of Veterinary Medicine Vienna (Pottenstein, Austria). The experiment lasted 7 weeks. A feeding model was used to induce transient SARA as following: 2 weeks baseline feeding (only forage, B), 1 week adaptation to SARA followed by 1 week SARA challenge (SARA 1, S1), 1 week challenge break (only forage, CB), followed by additional 2 weeks of SARA challenge (SARA 2, S2) (Figure 1A).

The forage-mix fed to cows during baseline and SARA challenges comprised 50% grass silage and 50% second-cut meadow hay on dry matter (DM) basis containing 54.4% DM, 91.6% organic matter (OM), 11.3% crude protein (CP), and 50.0% neutral detergent fiber (NDF). During the baseline and challenge break cows only received the forage mix diet with free access to water and mineral licking stones. Concentrate feed that replaced part of the forage in the diet to induce SARA mainly consisted of barley grain (33.0%), wheat (30.0%), corn (15.0%), and rapeseed meal (17.0%). Details of the analysis and chemical composition of dietary ingredients and SARA diet are given in Table S1 and in Pourazad et al. (2015). During the adaptation week the concentrate level was increased daily by 10% to reach 60% concentrate in the diet (DM basis) on day 7 (Figure 1A). During both challenges, the concentrate level was maintained at 60% level (DM basis). Cows were restrictively fed. During the baseline feeding and until day 4 of the adaptation to the high-concentrate diet, diet was offered at 1.5% of BW, whereas during the SARA challenges DM intake was set to 2.0% of BW. Forage mix and concentrate were offered separately in four and two feeding troughs, respectively, which were equipped with electronic scales and computer-regulated access gates (Insentec B.V., Marknesse, Netherlands) to control individual feed intake of each cow. Fresh feed was provided continuously. During SARA challenges, cows had daily access to the forage mix from 8 a.m. and to concentrate from 10 a.m. Cows that did not consume their complete concentrate allowance were force-fed the remaining concentrate through the rumen cannula. Detailed results regarding SARA model, ruminal pH dynamics, rumen temperature, short chain fatty acid dynamics, feed, and water intake were recently published in Pourazad et al. (2015).

### Ruminal pH measurements

To continuously monitor ruminal pH, each cow received a ruminal pH-sensor (smaXtec animal care sales GmbH, Graz, Austria) 4 days before the start of the feeding experiment; the pH sensors were validated recently (Klevenhusen et al., 2014). After calibration according to the company's instructions, sensors were manually inserted into the ventral rumen via the cannula. The sensors measured pH every 10 min and data were transmitted in real-time to the base station. The criterion for occurrence of SARA was duration of rumen pH below 5.8 for at least 300 min/d (Zebeli et al., 2008; Klevenhusen et al., 2014).

### Rumen papillae sampling

Rumen papillae sampling was done at day 1 (baseline, before concentrate was fed: B), day 15 (SARA challenge 1, after first SARA challenge: S1), day 26 (after the challenge break, CB), and day 37 (SARA challenge 2, after second and longer SARA challenge: S2), as shown in Figure 1A. Papillae biopsies (whole papillae of ~1 cm^3^, 0.5 g as triplicate) were taken from the ventral rumen wall directly under the rumen fistula with aseptic scissors and tweezers after the rumen was emptied and papillae were rinsed with sterile 1 × PBS, cut in small pieces and stored in RNA Later (Ambion, Life Technologies, Vienna, Austria) at −80°C for further analysis. The rumen was never empty for longer than 10 min.

### DNA extraction

The rumen papillae samples were thawed on ice and, after washing in 1 × PBS, genomic DNA was extracted from 0.25 g rumen papillae, using the PowerSoil DNA Isolation Kit (MO BIO Laboratories, Inc., California, USA) according to the manufacturer's protocol (http://www.mobio.com), with one modification (mechanical lysis for 20 min instead of 10 min). DNA concentration was determined using the Qubit 2.0 Fluorimeter (Qubit dsDNA BR Assay Kit, Thermo Fisher Scientific, Vienna, Austria). We screened the DNA extraction kit used for contamination before usage (by Illumina sequencing as described below). This no-template control resulted in 1122 reads of which the most abundant OTU detected had 90 sequences. Taxonomic classification of the no-template control-reads does not match with highly abundant OTUs found in the samples taken during our feeding experiment.

### Sequencing, sequence processing, and analysis

Amplicon sequencing was performed using Illumina MiSeq sequencing platform (Microsynth AG, Balgach Switzerland). The V345 hypervariable region of bacterial 16S rRNA genes was amplified using the primer set 341F (5′-CCTACGGGRSGCAGCAG-3′; Zakrzewski et al., 2012) and 909R (5′-TTTCAGYCTTGCGRCCGTAC-3′; Tamaki et al., 2011) to generate an approximate amplicon size of 568 bp. 16S rRNA gene PCRs, library preparation and sequencing was performed by Microsynth. Libraries were constructed by ligating sequencing adapters and indices onto purified PCR products using the Nextera XT Sample Preparation Kit (Illumina) according to the recommendations of the manufacturer. Equimolar amounts of each of the libraries were pooled and submitted for sequencing on an Illumina MiSeq Personal Sequencer using a 300 bp read length paired-end protocol. After sequencing the corresponding overlapping paired-end reads were stitched by Microsynth.

Sequence data were analyzed with the software package mothur (http://www.mothur.org/), according to the Illumina MiSeq protocol described previously (Kozich et al., 2013). Primer, barcode sequences and sequences of low quality and short length were trimmed with a minimum average quality score of 35 (default window size was used = 50 bp) and a minimum length of 500 bp. The command “Chimera.uchime” was used to exclude chimeric sequences. A total of 1,215,618 sequences (43.6%) passed the stringent quality control and were randomly subsampled to 40,000 sequences per sample. The sequences were clustered into operational taxonomic units (OTUs) with a cutoff of 97% 16S rRNA gene similarity (= 0.03 distance). For alignment the SILVA SSU NR reference database v119 (Pruesse et al., 2007) and for taxonomic classification the RDP trainset (trainset9_032012.rdp.tax) was used. The remaining 6833 OTUs were used for all downstream analysis. For calculation of the nonparametric species richness estimators Chao 1 and ACE, the diversity indices Shannon, Simpson and Shannoneven, the “summary.single” command was used. Heatmaps were created using JcolorGrid (Joachimiak et al., 2006). For Bray-Curtis analysis, Explicet version 2.10.15 (Robertson et al., 2013) was applied. The 50 most abundant OTUs of all sampling time points were additionally classified against type strains using the Greengenes database (http://greengenes.lbl.gov) (DeSantis et al., 2006) as well as by NCBI BLASTn against GenBank nr (http://blast.ncbi.nlm.nih.gov/Blast.cgi).

### Identification of indicator phylotypes

We compared the 500 most abundant OTUs which showed significant changes in relative abundance from B to S1, CB, and S2 and combined the results at the genus level. Then, we divided genera with decreasing abundance from genera with increasing abundance during SARA challenges and challenge break.

### qPCR of key phylotypes

The abundance of the three OTUs that were significantly shifted during SARA challenges (OTU 2, OTU 5, and OTU 9) was confirmed by qPCR approaches on a Stratagene Mx3000P real-time PCR System (Agilent Technologies, Santa Clara, USA) and additionally 10 most prevalent OTUs (relative abundance ≥1%) were analyzed as pooled samples by sampling time point. Results were analyzed using the associated software (Stratagene MxPro, QPCR Software, version 2.00). DNA samples were assayed in duplicate in a 20 μl reaction mixture containing 10 μl 2 × Brilliant III Ultra-Fast SYBR Green qPCR Master Mix (Agilent, Vienna, Austria), 2 μl of each primer (2.5 μM), 5 μl of nuclease-free water, and 1 μl DNA template (2–50 ng/μl). Amplification involved one cycle at 95°C for 3 min and 40 cycles of 95°C for 5 s followed by 20 s at 61°C and 57°C for general bacteria and OTU-specific primer-pairs, respectively. A melting curve ranging from 70 to 90°C, with fluorescence measurements at 1°C intervals, was done after all real-time PCRs. To determine the specificity of the reaction, an *in-silico* PCR using TestPrime (http://www.arb-silva.de/search/testprime/) against the SILVA database was done and no non-target match was found for any of the primers designed in this study. Confirmation of the primers designed for this study was done by Sanger-sequencing the qPCR amplicons produced by each primer pair. The standard for qPCR was prepared with pooled DNA from all samples and serial dilutions of the purified PCR products were used for standard curves as described previously by Li et al. (Li et al., 2009) using the primer panel 27F (forward = 5′- AGAGTTTGATYMTGGCTCAG -3′) and 1492R (reverse = 5′-GGYTACCTTGTTACGACTT-3′) (Lane, 1991). For OTU-specific standard curves, purified qPCR products with each of the OTU-specific primer pairs (Table S2A) of a mixed DNA sample consisting of 1 μl DNA of each sample were used with the same thermal protocol as described below. Standard curves (range: 1e+3–1e+7 gene copy numbers) were included in each qPCR assay. Negative controls were included in duplicates. Details regarding evaluation of primer pairs designed in this study are given in Table S2B.

### Statistics

Data were checked for normal distribution before analysis by applying the Kolmogorov-Smirnov-Test implemented in IBM SPSS (version 22, IBM Vienna, Austria). Statistical analysis for detecting significant changes on OTU and phylum level regarding SARA challenge was done using the parametric Oneway ANOVA with Tukey test implemented in IBM SPSS. The model included the fixed effect of SARA challenge (B vs. S1 vs. CB vs. S2). Differences in species richness and diversity indices were examined by Oneway ANOVA with Tukey test using IBM SPSS. The level of significance was set at *P*-values of 5% (*P* ≤ 0.05), whereas 0.05 < *P* ≤ 0.10 was defined as a trend. Linear discriminant analysis and dendrogram (ward method) were computed with OTUs that occur in all sampling time points (*n* = 270), using JMP®; (Version 10.0.0, SAS Institute Inc., Cary, NC).

To analyze potential co-occurrences between OTUs within each sampling time point (B, S1, CB, S2) we considered the mathematical and topological features of the pairwise Spearman correlations (*r*_*s*_) between the 50 most abundant OTUs using R statistical computing environment (R Development Core Team, 2015. Foundation for Statistical Computing, Vienna, version 3.1.3, http://www.r-project.org). The resulting correlation matrix of the 50 most abundant OTUs was converted into a network. In the next step, the graph was visualized as an arc-diagram (http://gastonsanchez.com/software/arcdiagram_introduction.pdf, 2015), with self-loops being removed. Strong correlations |*r*_*s*_| ≥ 0.7 between OTUs for each sampling time point were visualized as an arc-diagram. In addition, we visualized the correlation matrix (method: “original”) over all sampling time points (https://cran.r-project.org/web/packages/corrplot/corrplot.pdf). The calculations and visualization were implemented in R statistical computing environment (R Development Core Team, 2015. Foundation for Statistical Computing, Vienna, version 3.1.3, http://www.r-project.org).

qPCR data was tested for normal distribution using the Kolmogorov-Smirnov-Test implemented in IBM SPSS (version 22, IBM Vienna, Austria). The Wilcoxon Test was applied to find significant shifts between sampling time points and the Bonferroni test was used to correct for multiple comparisons.

### Accession numbers

Sequencing data are available in BioProject SRA database under the accession number PRJEB9353.

## Results

### Intensity of SARA and ruminal pH

Cows were clinically healthy throughout the feeding experiment. Duration of pH <5.8 at B (baseline) was 0 min/d for each of the eight experimental cows. During S1, time of pH <5.8 min/d increased differently for the cows, with three cows experiencing SARA and the remaining five cows having decreased ruminal pH but still above the SARA threshold of 300 min/d of pH <5.8. In the challenge break CB, ruminal pH recovered from SARA conditions in all cows except for one cow, whose duration of ruminal pH <5.8 was 240 min/d at the day before sampling time point CB (Table S3). All cows experienced SARA at S2. Two cows had a significantly lower average ruminal pH (*P* < 0.05) during both SARA challenges (Table S3).

### Epimural microbiome composition

**Figure 2:**
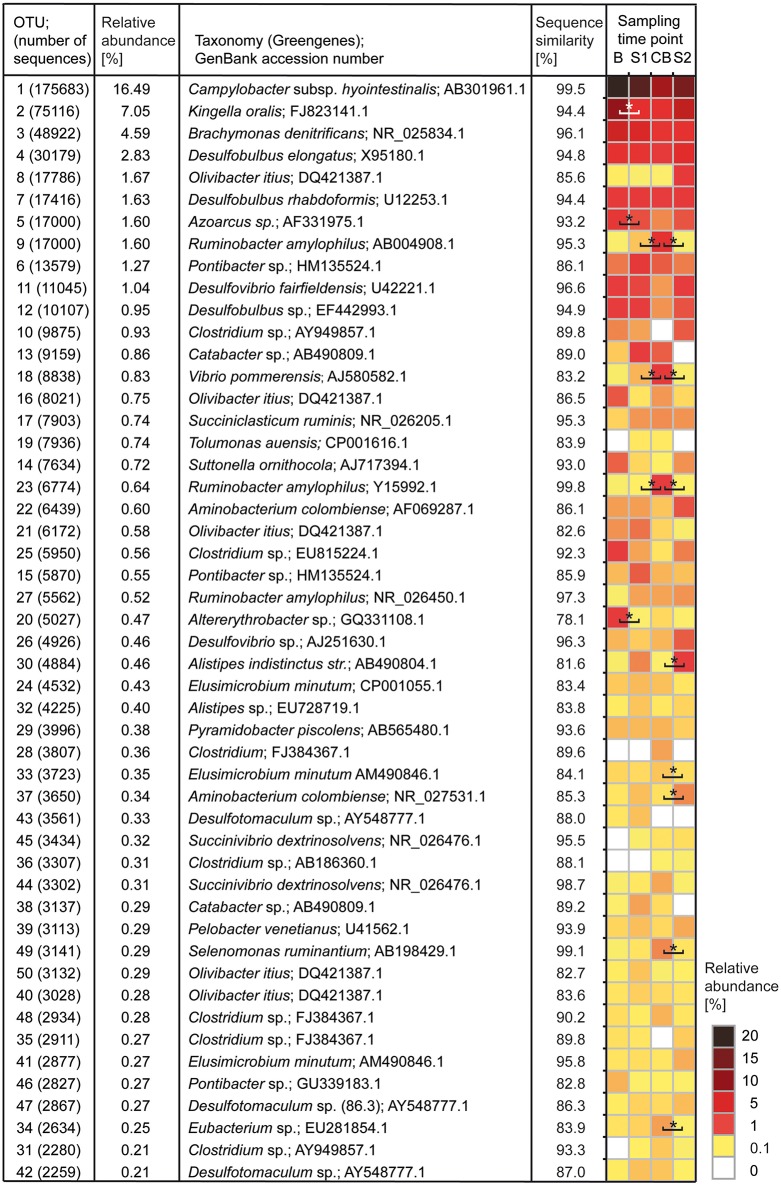
**Heatmap showing the 50 most abundant OTUs with relative abundance and best Greengenes type strain hit**. OTUs are sorted according to relative abundance. Statistically significant shifts between two sampling time points are marked with an asterisk. Sampling time points are as follows: B (baseline), S1 (SARA 1), CB (challenge break), and S2 (SARA 2).

All 2,789,089 reads deriving from 32 samples were processed together. In total 1,215,618 sequences (43.6%) with a minimum length of 500 base pairs remained after a stringent quality control and subsampling (37,988 ± 2028 sequences per sample) and were clustered into 200,339 OTUs, from which 193,506 OTUs were excluded because they contained <10 sequences. The remaining 6833 OTUs were used for all further downstream analyses. In total, 19 phyla were identified with *Firmicutes, Proteobacteria*, and *Bacteroidetes* being most abundant with 96.1% of all reads affiliating to these phyla. Next abundant phyla were *Synergistetes* and *Elusimicrobia*. OTU 1, with 16.5% relative abundance, was classified as *Campylobacter hyointestinalis* with 99.5% sequence similarity to the best Greengenes type strain hit. OTU 2 (7.1% relative abundance) was classified as *Kingella oralis* with 94.4% sequence similarity to the best Greengenes type strain hit. OTUs 3, 4, 8, and 7 were classified as *Brachymonas dentrificans* (4.6% relative abundance, 96.1% sequence similarity), *Desulfobulbus elongatus* (2.8% relative abundance, 94.8% sequence similarity), *Olivibacter itius* (1.7% relative abundance, 85.6% sequence similarity), and *Desulfobulbus rhabdoformis* (1.6% relative abundance, 94.4% sequence similarity), respectively. In total, ten OTUs showed more than 1% relative abundance (Figure 2). Additionally, the 50 most abundant OTUs were classified against type strains and for their best BLASTn hit against GenBank nr (Table S4). In total 299 genera were detected with *Campylobacter* being the most abundant (16.3% relative abundance). The second most abundant genus was *Kingella* (7.2% relative abundance), followed by *Desulfobulbus* (5.1% relative abundance), *Brachymonas* (5.0% relative abundance), *Pseudosphingobacterium* (4.4% relative abundance), and *Alkalibaculum* (3.6% relative abundance). Summed up, 25 genera showed more than 1% relative abundance (Table S5).

### Time point-related shifts in BEBM

Diversity indices and evenness estimators differed significantly between sampling time points. Species richness was significantly higher at CB compared to the B, S1, and S2. Shannon diversity index also increased at CB, Simpson diversity index decreased from B to CB and increased at S2 and evenness decreased at CB compared to B and S2 (Table 1).

**Table 1:** **Species richness, diversity and evenness indices of epimural bacteria are depicted for sampling time points B (baseline), S1 (SARA 1), CB (challenge break), and S2 (SARA 2)**.

	**Sampling time point**				**SEM^1^**	***P*-value**
	**B**	**S1**	**CB**	**S2**		
Coverage	0.91^A^	0.91^a^	0.88^bB^	0.92^a^	0.004	0.003
Chao1	11,833^b^	12,367^b^	16,830^a^	12,168^b^	547	0.008
ACE^2^	22,787^b^	23,768^b^	33,634^aA^	24,500^B^	1121	0.009
Shannon	5.00^b^	5.31^b^	6.18^a^	5.00^b^	0.103	0.001
Simpson	0.07^Aa^	0.04^B^	0.02^b^	0.04^a^	0.004	0.001
Shannoneven	0.60^b^	0.64^B^	0.72^aA^	0.62^b^	0.010	0.001

*Significant differences (between sampling time points) in rows are marked with different letters*.

1 *SEM = standard error of the mean*.

2 *ACE = abundance-based coverage estimator*.

**Figure 3:**
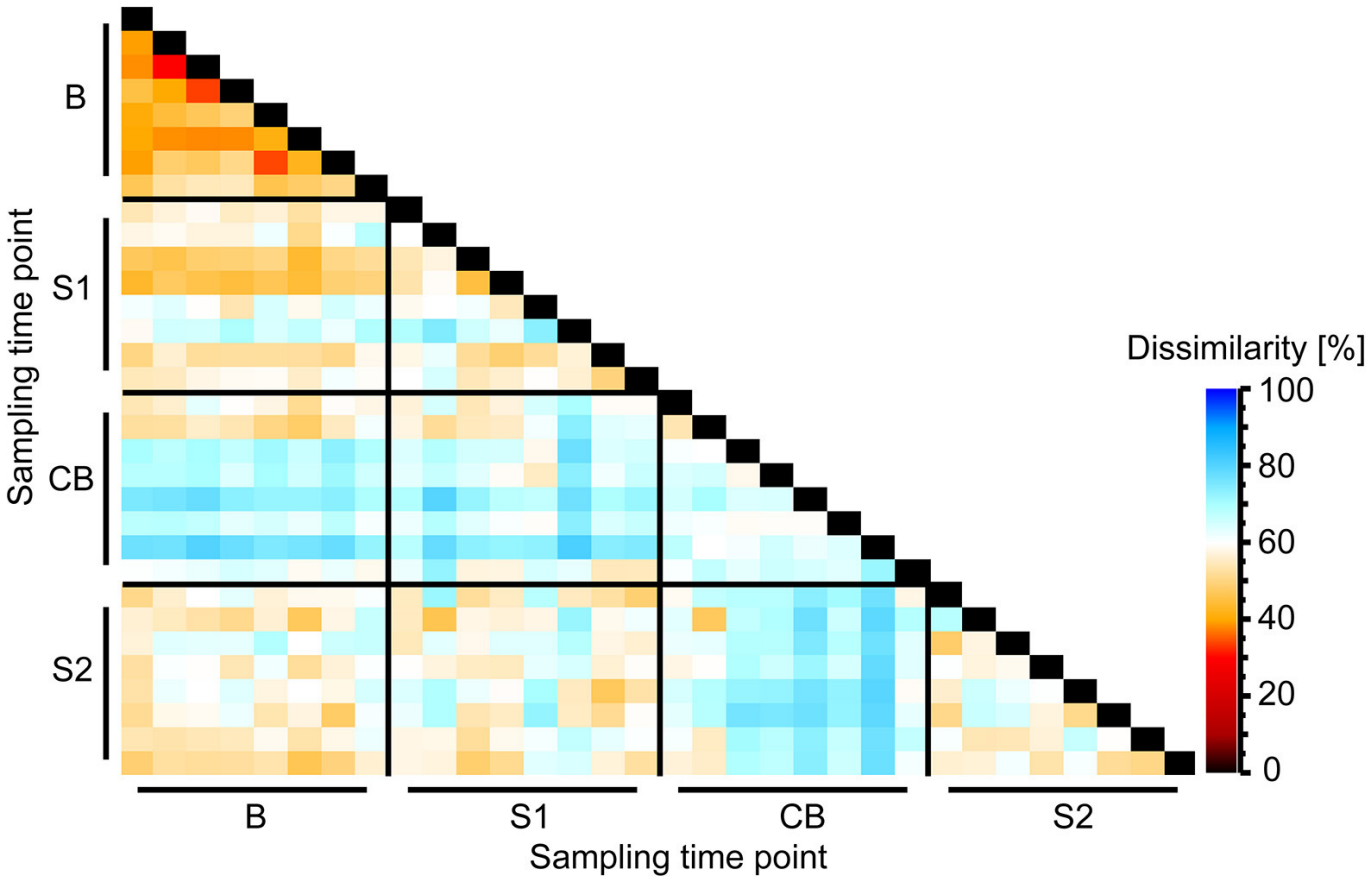
**Heatmap showing Bray-Curtis dissimilarity between bacterial epimural communities between different sampling time points B (baseline), S1 (SARA 1), CB (challenge break), and S2 (SARA 2)**.

On community level—shown by Bray-Curtis analysis—the samples taken at B were more similar among cows than samples taken at S1, CB, and S2. On the other hand, the samples taken at CB differed most among cows compared with other sampling time points. Samples taken at CB also differed most from samples taken at all other sampling time points (Figure 3). Linear discriminant analysis also revealed distinct clustering for the BEBM at different sampling time points (Figure 1B). The dendrogram showed distinct clustering for B and S2 while S1 and CB did not cluster separately (Figure 1C). In total, 20% of the 50 most abundant OTUs showed significant differences between at least two different sampling time points, e.g., OTU 2, OTU 5, and OTU 9, classified as *Kingella, Azoarcus*, and *Ruminobacter*, respectively.

In Figure S1, relative abundances of phyla are shown for each sampling time point and *P*-values are shown in Table S6. Summarized, *Proteobacteria, Spirochaetes, Lentisphaerae*, and *Deinococcus-Thermus* decreased significantly from B to S1 and there was a trend of decreasing *Deferribacteres* from B to S1. *Tenericutes* decreased significantly and there was a trend of decreasing *Firmicutes* from CB to S2. In B and S1 *Proteobacteria* were numerically more abundant than *Firmicutes* while this ratio changed in CB and S2.

In B, three OTUs (*Campylobacter*-OTU 1, *Kingella*-OTU 2, and *Brachymonas*-OTU 3) showed a relative abundance >5% (Table S7). In S1 and CB only OTU 1 (*Campylobacter*) was highly abundant with a relative abundance >5%. In S2, OTU 1 (*Campylobacter*) and OTU 2 (*Kingella*) had a relative abundance >5%. OTUs 2, 5, and 20 decreased significantly during the feeding experiment and OTUs 9, 18, and 23 were significantly increased in CB compared with the other sampling time points. OTUs 22, 30, 34, 37, and 49 also showed significant shifts in relative abundance during the feeding experiment. Changes by trend were seen for OTUs 1, 26, 42, and 47.

### Correlations between ruminal pH and OTU abundances

**Figure 4:**
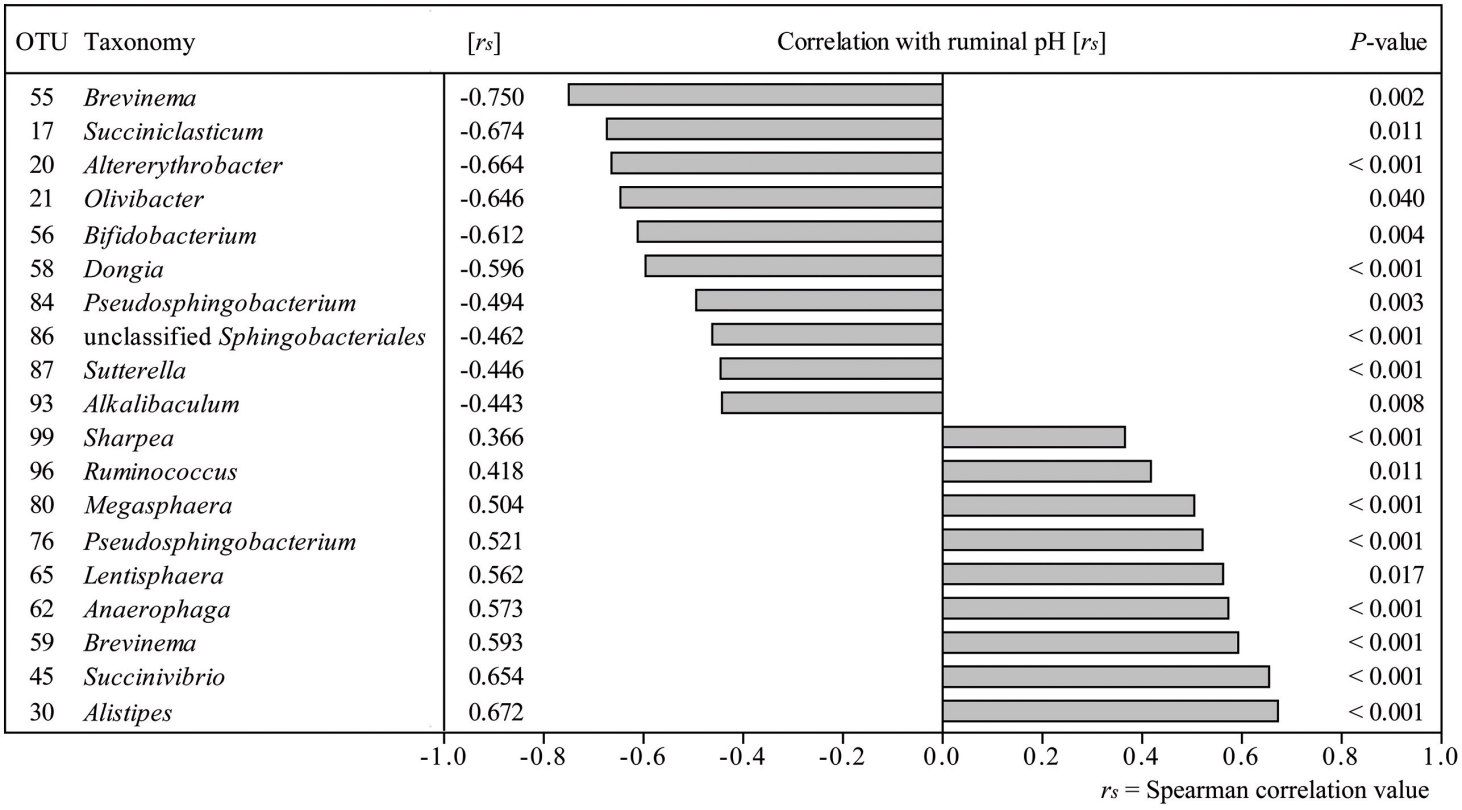
**Correlation analysis of 100 most abundant OTUs with ruminal pH over all sampling time points**. Only significant correlations are depicted.

Correlation analysis of 100 most abundant OTUs with average ruminal pH per sampling time point revealed 19 OTUs, which correlated significantly (*P* < 0.05) with ruminal pH (Figure 4). Strongest positive correlations with ruminal pH were found for OTU 30 (*Alistipes*), OTU 45 (*Succinivibrio*), and OTU 59 (*Brevinema*) and strongest negative correlations were found for OTU 20 (*Altererythrobacter*), OTU 17 (*Succiniclasticum*), and OTU 55 (*Brevinema*). Correlation analysis results were confirmed by additionally computing correlations between qPCR results and average ruminal pH (Table S8).

### Correlation among the 50 most abundant OTUs

**Figure 5:**
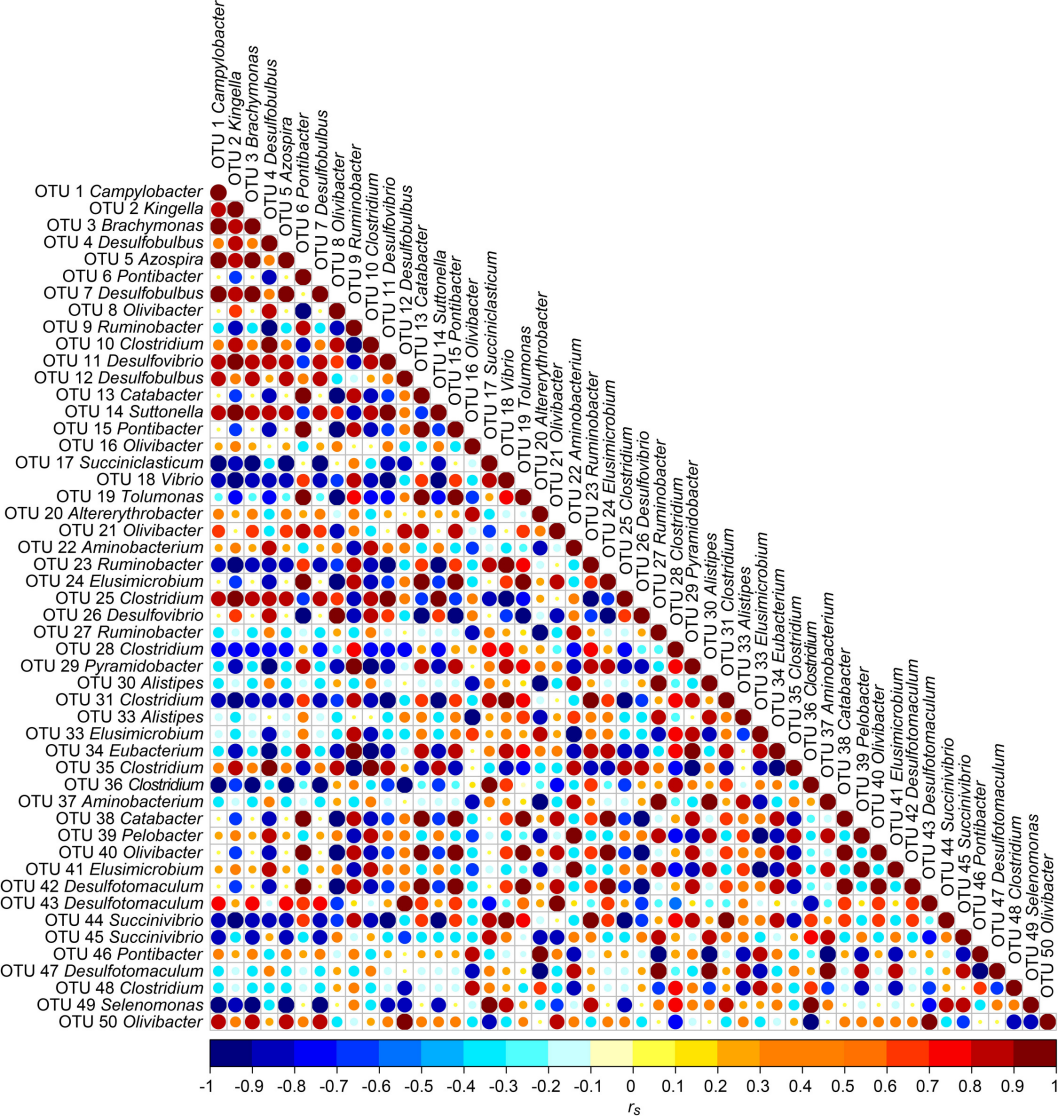
**Correlation matrix between the 50 most abundant OTUs over all four sampling time points B (baseline), S1 (SARA 1), CB (challenge break), and S2 (SARA 2): Positive correlations are shown in red and negative correlations in blue**. Color intensity and the size of the dots are proportional to the correlation values [*r*_*s*_] within a correlation group.

Correlation analysis between the 50 most abundant OTUs was done to analyze potential co-occurrences between OTUs and results are displayed in a correlation matrix (Figure 5). In order to analyze possible co-occurrences in more depth, the relationship between the OTUs within each sampling time point were investigated. In this context, 395 correlations (8.2%) were highly positively or negatively correlated (|*r*_*s*_| ≥ 0.7), which are shown in Figure S2. In B 2.1%, in S1 1.9%, in CB 2.5%, and in S2 1.7% from a total of 4812 correlations were highly correlated. Strongest positive correlations were found between OTU 6 and OTU 15 (both *Pontibacter*) for B, CB, and S2 (|*r*_*s*_| > 0.97; *P* < 0.001). For S1, strongest positive correlations were found between OTU 10 and OTU 35 (both *Clostridium*) (|*r*_*s*_| > 0.97; *P* < 0.001). Strongest negative correlations were found between OTU 1 (*Campylobacter*) and OTU 38 (*Catabacter*) in B (|*r*_*s*_| > 0.93; *P* < 0.001), between OTU 2 (*Kingella*) and OTU 11 (*Desulfovibrio*) in S1 (|*r*_*s*_| > 0.92; *P* < 0.001), between OTU 31 (*Clostridium*) and OTU 44 (*Succinivibrio*) (|*r*_*s*_| > 0.92; *P* < 0.001) in CB, and between OTU 11 (*Desulfovibrio*) and OTU 35 (*Clostridium*) in S2 (|*r*_*s*_| > 0.90; *P* < 0.003).

### Indicator phylotypes for non-SARA conditions and SARA conditions

**Figure 6:**
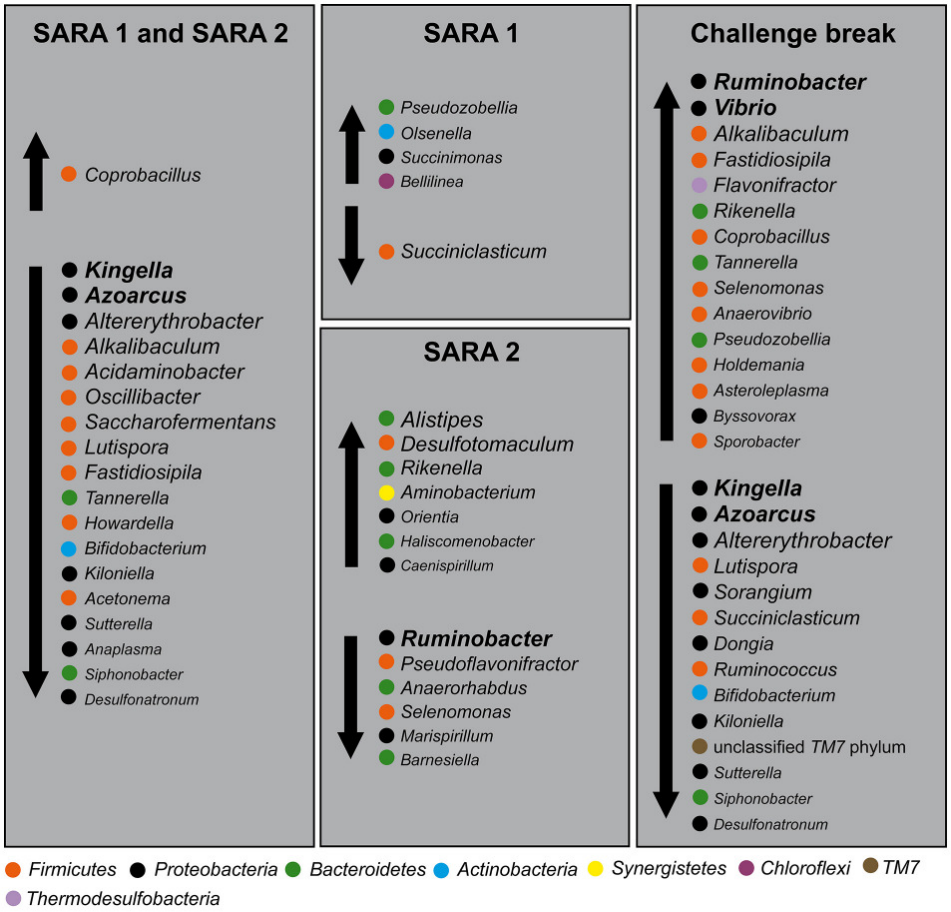
**Putative indicator phylotypes for non-SARA and SARA conditions**. Genera with significant shifts within SARA 1, SARA 2, and challenge break were grouped regarding their change in abundance under SARA conditions. Vertical arrows indicate an increase or decrease with SARA challenge or challenge break. Phyla are indicated with colored dots. Abundance ranking of taxa is indicated by font size (the larger, the more abundant).

Figure 6 shows several putative indicator taxa for non-SARA conditions (*Kingella, Azoarcus, Altererythrobacter, Alkalibaculum, Acidaminobacter, Oscillibacter, Saccharofermentans, Lutispora, Fastidiospila, Tannerella, Howardella, Bifidobacterium, Kiloniella, Acetonema, Sutterella, Anaplasma, Siphonobacter*, and *Desulfotonatronum*) and SARA conditions (*Coprobacillus*), which changed in abundance significantly from B to S1 and S2. In total, 24 genera decreased and 12 genera increased with SARA challenges. Interestingly, 19 genera showed the same statistically significant shifts during both SARA challenges (listed above), five genera only changed with S1 and 13 genera only changed with S2. During the challenge break 29 genera showed significant shifts, of which 15 genera increased and 14 genera decreased.

### qPCR results confirmed sequencing data

**Figure 7:**
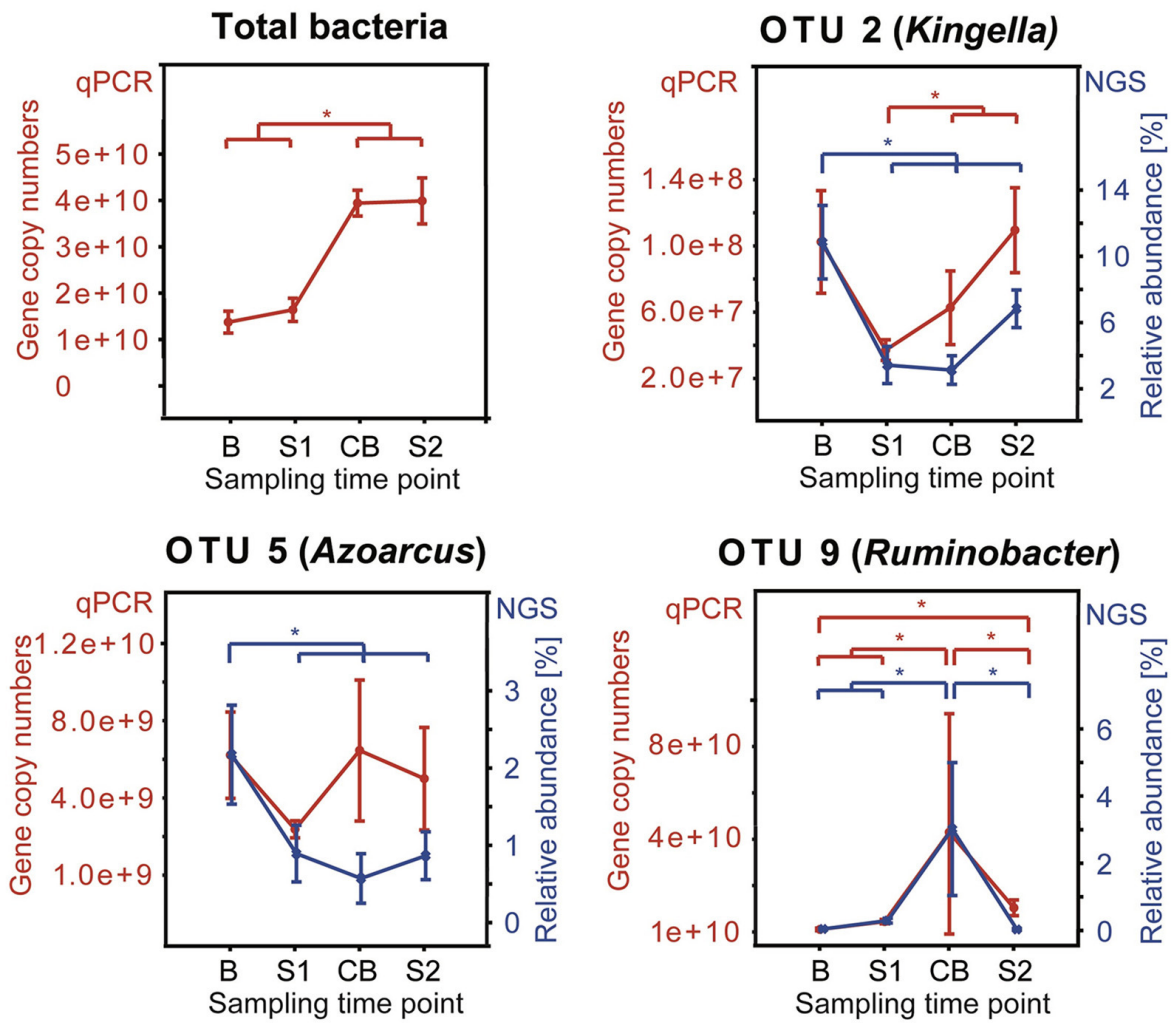
**Comparison between qPCR results and sequencing data (NGS) for OTUs showing relative abundance >1% and significant shifts during the feeding experiment**. Median of each sampling time point is shown with standard error. qPCR results are shown as copy numbers per gram rumen papillae in red and NGS results are shown as relative abundance in blue. Sampling time points are as follows: B (baseline), S1 (SARA 1), CB (challenge break), and S2 (SARA 2). An asterisk indicates a significant shift.

qPCR analysis showed a total 16S rRNA gene abundance of 1.4 × 10^10^, 1.6 × 10^10^, 3.9 × 10^10^, and 4.0 × 10^10^ gene copy numbers per gram rumen papillae in sampling time points B, S1, CB, and S2, respectively. OTUs (OTU 2, OTU 5, and OTU 9) with more than 1% relative abundance that showed significant shifts over sampling time points in the MiSeq sequencing approach were analyzed by qPCR and the results largely confirmed our sequencing results, albeit some shifts in the qPCR data were not statistically significant (Figure 7). Both, OTU 2 and OTU 5 decreased from B to all other sampling time points. OTU 9 increased from S1 to CB and decreased from CB to S2. High abundance of the 10 most abundant OTUs was confirmed and at least 10^6^ copy numbers were detected as mean of each sampling time point (data not shown).

## Discussion

### Intensity of SARA and changes in ruminal pH

During CB, ruminal pH of all cows—except for one cow—recovered completely from SARA challenge, which indicates that 1 week break sufficed to recover rumen pH from SARA conditions. In S2, all cows experienced SARA, which, contrary to our hypothesis, was characterized by stronger decline of pH compared with S1 challenge. It seems that cows respond differently to the first SARA challenge despite of the same diet. During S2 the inter-individual pH variation was less pronounced. The response of pH to diet depends also on how cows modulate their feed intake in particular diurnal intake of forages as a self-prevention strategy against SARA, as described recently (Hendriksen et al., 2015) and feed intake data of this study is published in Pourazad et al. (2015). The mechanisms behind this behavior and different susceptibilities of cattle to SARA are not yet fully understood but differences in bacterial community composition in the rumen as well as to host genetic factors are recently discussed to play a role (Humer et al., 2015). Indeed, there is an increasing interest in understanding the mechanisms behind different susceptibilities to SARA, in particular to understand the role of ruminal bacterial changes associated with SARA, which would help developing more effective preventative strategies against SARA in cattle (Penner et al., 2009; Chen et al., 2012; Schlau et al., 2012). Because of the self-prevention strategy, where cows sort feed mainly in favor of fiber-rich feed particles, SARA appears as an intermittent chronic disorder, which we mimicked with the transient SARA model in this study. To our best knowledge, this is the first study to represent dynamic BEBM responses to a typical, chronically intermittent SARA.

### Structure of epimural bacterial microbiome

The dendrogram showed distinct clustering for B and S2 while S1 and CB partly overlap, indicating that at CB, the BEBM composition did not re-establish the baseline conditions after the first and short SARA challenge in contrast to ruminal pH which recovered fully. This is consistent with results from experiments in which rumen contents of cows having different pH values were swapped, and the pH returned to pre-swap values very quickly but the rumen bacterial community took much longer to return to its original composition (Weimer et al., 2010). The high instability and disarrangement of the BEBM composition at the CB shows that a 1-week break from the challenge is not sufficient to re-establish the baseline conditions; it might rather represent a still ongoing adaptation process. This unstable composition seems to prolong adaptation processes of the BEBM during S2 and likely to negatively influence the stability of ruminal pH during S2. Petri et al. (2013) found that samples taken from bulls fed high-forage diets clustered more closely compared to samples taken from high-grain diet fed bulls, which is in accordance with our results. This confirms that a SARA challenge disturbs and unbalances the microbiome composition. A significantly increased diversity during CB shows that the BEBM composition was highly affected during CB, which seems to be caused by the first SARA challenge, leading to a severe instability during CB. A decrease in ruminal pH might affect several bacterial groups, and increase potentially harmful, acid-resistant phylotypes (Khafipour et al., 2009). Bacterial indicator phylotypes for non-SARA conditions (e.g., *Kingella, Azoarcus*, and *Altererythrobacter*) and SARA conditions (e.g., *Coprobacillus*) found in our study provide proof for specific shifts in the BEBM, which are directly linked to changes in the diet over time.

In our study, significant shifts in BEBM composition over time indicate a high impact of dietary changes on epimural bacteria. However, existing differences in highly abundant OTUs among different studies might be due to different origin of the animals and management (Petri et al., 2013). High abundant phylotypes might be replaced by other phylotypes that take over the same function in the bacterial community (Taxis et al., 2015). Geographical differences could possibly affect establishment of specific, highly abundant bacterial groups for different cattle populations, which might be caused by contact with geographical-specific phylotypes. A recent study showed multiple microbial species can fulfill the same function, with different combinations of microbes being highly abundant depending on the diet (Henderson et al., 2015).

### Highly abundant key OTUs affected by SARA and indicator phylotypes

With average read lengths of 550 bp, reads from Illumina MiSeq sequencing can provide only limited phylogenetic resolution and therefore the “best hits” to species level provided in this study should be interpreted with caution. Therefore, when talking about taxonomic identification of the OTUs, we refer to the genus level. The most abundant OTU in our study was classified as *Campylobacter*. *Campylobacter* was detected in high numbers in the bacterial epimural community of cattle (Zhao et al., 2014) and of goats before (Jiao et al., 2015; Wetzels et al., 2015). However, this is the first study which revealed *Campylobacter* being most abundant in the BEBM in adult dairy cows. The function of *Campylobacter* in the rumen is not known yet, although some studies suggest a possible involvement in ammonia metabolism (Jiao et al., 2015). For cows, only some *Campylobacter* species are known to cause diseases, like enteritis (*C. jejuni* and *C. coli*) while some are regarded as commensals (*C. hyointestinalis*) in the rumen (Li et al., 2012) and feces (Guevremont et al., 2014). However, the high abundance of *Campylobacter* in the BEBM of asymptomatic cows leads to the hypothesis that *Campylobacter* detected in our study are non-pathogenic to the host and might be involved in nitrogen metabolism. The second most abundant OTU was classified as *K. oralis* which was detected in rumen samples before (Nagaraja et al., 1978). The significant decrease of *Kingella*-OTU 2 from B to S1, CB, and S2 is in accordance with two recent studies, where closely related *Neisseriaceae* changed similarly at the rumen epithelium in growing goats (Jiao et al., 2015; Wetzels et al., 2015). OTU 2 might thus be promoted by increasing amounts of easily fermentable carbohydrates in the high-concentrate diet. OTU 5 showed similar changes in relative abundance as OTU 2. *Azoarcus* sp. (OTU 5) has not been detected in rumen samples before and is mostly known as a nitrogen fixing bacterium from marine water, grasses and from termite gut samples (Desai and Brune, 2012; Fernandez et al., 2014). *Azoarcus* in the rumen might fulfill the same function as in the termite gut, where it compensates for the lack of nitrogen in the diet (Desai and Brune, 2012). Changes in dietary protein content could possibly be responsible for changes in abundance of OTU 5. qPCR data for OTU 5 show an opposite trend (although not statistically significant) than sequencing data during CB and S2, which might be explained by the high variation between the cows at these sampling time points. The correlation pattern of the latter OTUs with other highly abundant OTUs and the ruminal pH were also similar. Interestingly, *Ruminobacter amylophilus* (OTU 9) increased from S1 to CB and decreased in S2. These results are in contrast to the current state of knowledge that *R. amylophilus* is amylolytic and proteolytic in the rumen; and is not pH sensitive (Cheng and McAllister, 1997). Thus, OTU 9 was expected to increase during the SARA challenge. Chen et al. (2011) found *Ruminobacter* to be highly abundant in the ruminal BEBM in steers. The abundance of *Ruminobacter* was positively correlated with dietary starch intake, which could not be confirmed by our results. Other yet unknown factors than ruminal pH or starch- and protein content in diet could affect the abundance of OTU 9 in the BEBM. Further OTUs respond differently to the SARA conditions in the rumen and their respective best Greengenes hits are known for various different functions like amino acid degradation (*Vibrio*-OTU 18*, Aminobacterium*-OTU 22 and −37*, Eubacterium*-OTU 34) (Kafkewit and Goodman, 1974; Baena et al., 2000), glucose metabolism (*Alistipes*-OTU 30*, Selenomonas*-OTU 49) (Bryant, 1956; Hungate, 1966; Nagai et al., 2010), sulfate-reduction (*Desulfotomaculum*-OTU 42) (Spring et al., 2012) and fiber fermentation by cross-feeding processes with other microbes (*Selenomonas*-OTU 49) (Sawanon et al., 2011). The ability to use a variety of energy sources indicates the versatility of some organisms (*Eubacterium*-OTU 34) (Genthner et al., 1981) and makes it difficult to interpret their metabolic functions in the BEBM. Some of these phylotypes have not or only sporadically been detected in the rumen before (*Altererythrobacter*-OTU 20*, Alistipes*-OTU 30) (Reti et al., 2013), while the others are well-known members of the rumen microbial community (Hungate, 1966; Wallace et al., 2003; Fernando et al., 2010). Strikingly, we found high amounts of sulfate-reducing bacteria (e.g., *Desulfovibrio, Desulfotomaculum*) (7.7%), which is in contrast to literature, where sulfate-reducing bacteria were detected in low relative abundances in rumen samples (Pei et al., 2010; Spring et al., 2012). Sulfate-reducing bacteria can also use nitrate, fumarate, acetate, and other organic compounds as substrate, which impedes the prediction of their metabolic potential in the rumen (Muyzer and Stams, 2008).

OTUs with the same correlation partners as OTUs 2, 5, and 9 might be important phylotypes regarding SARA conditions because abundance of the latter OTUs changed significantly during our feeding experiment. Eleven out of the 15 most abundant OTUs showed similar correlation patterns in the correlation matrix. Most correlations were highly positive (*r*_*s*_ > 0.7). We assume that highly abundant OTUs play a particularly important role in the bacterial community, since their abundance accounts for almost half of all sequences in our study (15 most abundant OTUs: 43.8%). When rumen conditions were shifted toward SARA conditions, the relative abundance of highly abundant OTUs decreased (e.g., *Altererythrobacter*-OTU 20) and the abundance of some lower abundant OTUs increased (e.g., *Coprobacillus-*OTU 288). Between closely related OTUs strong positive correlations were found, which also indicates that they might have the same function and react similarly to pH shifts. Strong positive correlations were also found between distantly related OTUs (Flint et al., 2008) what might be caused by cross-feeding effects.

Indicator phylotypes that shifted with SARA are known for a variety of metabolic functions and optimum pH needs. Therefore, the mechanisms behind shifts in BEBM could be different to those in the rumen content, where mainly fiber, starch, protein level (Belanche et al., 2012), and pH affects bacterial community composition (Nikkhah, 2012). Both changes in ruminal pH and in the BEBM proved the second and longer SARA challenge S2 to be much more severe than the first and shorter SARA challenge S1. This finding has particular implication in the grain feeding in dairy cattle such as during transition period from pregnancy to the onset of lactation (Humer et al., 2015). Our data recommend slow and long periods of feeding to help proper adaptation of BEBM, though avoiding grain feeding interruptions.

## Conclusion

Unlike ruminal pH which recovered fully after the first SARA challenge, results of this study indicate that BEBM could not re-establish baseline conditions during 1 week break. More interestingly, the study indicated that a 1-week break from the challenge of a high-grain diet changes the profile of the BEBM and thus might have negatively affected rumen function in the following 2-week high-grain challenge period, which was expressed with a stronger decline of ruminal pH and greater severity of SARA. Our results suggest that the adaptation to a high-grain diet needs to be slow and, more importantly, without interruption to allow the BEBM to cope with the dietary change. However, the role of the luminal bacterial community in the rumen also needs to be linked to these results, since the microbes in the rumen content may be just as important in dietary adaptation. Further studies are warranted to aid in the identification and function of the BEBM during transient SARA experiments, and also to verify these results which might help in shaping new feeding strategies in dairy farming.

## Author contributions

Peformed experiments: SW, EM, PP, MQ, FK. Analyzed data: SW, EM, BMZ, BP, MW, QZ, SSE. Conceived and designed experiments: QZ, MW, SSE. Wrote the paper: SW, EM, BMZ, BP, QZ, SSE.

## Funding

Funding for this research was provided by project “D-I.INFLACOW, LS12-010” of Vienna Science and Technology fund (WWTF) leaded by QZ. EM was funded by the science funds of the Land Niederoesterreich, Austria (project ID: MIKRORIND).

### Conflict of interest statement

The authors declare that the research was conducted in the absence of any commercial or financial relationships that could be construed as a potential conflict of interest.

## Abbreviations

BEBM: bovine epimural bacterial microbiome
B: sampling time point 1 (Baseline)
S1: sampling time point 2 (SARA 1)
CB: sampling time point 3 (Challenge break)
S2: sampling time point 4 (SARA 2).
