# Supplementary material for: Epimural Indicator Phylotypes of Transiently-Induced Subacute Ruminal Acidosis in Dairy Cattle
Source: Front Microbiol. 2016 Mar 4;7:274. doi: 10.3389/fmicb.2016.00274 (PMC4777738; doi:10.3389/fmicb.2016.00274)
Supplement: Supplementary file 1 [file Table1.PDF]

Table S1. Diet composition used in the feeding experiment.

Ingredient	Dry matter (DM) basis (%)
**Forage-mix composition (Baseline diet)[1]**	
Grass silage	50.0
Second-cut meadow hay	50.0
**Concentrate-mix composition[2]**	
Barley grain	33.0
Wheat	30.0
Corn	15.0
Rapeseed meal	17.0
Dried beet pulp	3.2
Calcium carbonate	0.5
NaCl	0.3
Mineral-vitamin premix[3]	1.0
**SARA-challenge diet**	
DM (% of fresh diet)	74.1
Organic matter	94.1
Crude protein	15.4
Neutral detergent fiber	31.8
Acid detergent fiber	19.9
Ether extract	1.71
Non-fiber carbohydrates	45.3

[1]The forage mix contained 54.4% DM, 91.6% organic matter, 11.3% crude protein, and 50.0% neutral detergent fiber (DM basis).

[2]Concentrate mix contained 87.3% DM, 95.8% organic matter, 18.1% crude protein, and 19.5% neutral detergent fiber (DM basis).

[3]Vitamin-mineral mix contained (g/kg): Ca, 220; P, 60; Mg, 30; Na, 60; Zn, 3; Mn, 5; I, 0.01; Se, 0.04; Co, 0.03; Cu,0.75; Vitamin A, 600.000 (IU); Vitamin D, 80.000 (IU), Vitamin E, 2.
