# Supplementary material for: Epimural Indicator Phylotypes of Transiently-Induced Subacute Ruminal Acidosis in Dairy Cattle
Source: Front Microbiol. 2016 Mar 4;7:274. doi: 10.3389/fmicb.2016.00274 (PMC4777738; doi:10.3389/fmicb.2016.00274)
Supplement: Supplementary file 2 [file Table2.PDF]

Table S2A. Primers used for qPCR.

OTU/ Target group	Sequences of primers and probes (5' - 3')	AT1	SYBR Standards RSq	SYBR, Y	Efficiency [%]	Linear dynamic range tested	Amplicon size (bp)	Reference
All bacteria	CCTACGGGAGG CAGCAG	61	0.998	-3.406*LOG(X) + 39.00	100.9	5.74E+03	189	Muyzer et al., 1993
	ATTACCGCGGCT GCTGG					5.74E+06		
OTU 1 *Campylobacter*	GGATGACACTTT TCGGAG	57	0.999	-3.747*LOG(X) + 37.89	85.1	3.52E+03	246	Rintillä et al., 2004
	AATTCCATCTGC CTCTCC					3.52E+06		
OTU 2 *Kingella*	CGCGTTAGCTAC GCTACTGA	57	0.999	-4.097*LOG(X) + 44.13	76.0	5.12E+03	239	this study
	CGTGCATTTGGA ACTGGTCA					5.12E+06		
OTU 3 *Brachymonas*	ATGCAGTTCCCA GGTTAGGC	57	0.998	-3.488*LOG(X) + 38.49	93.5	7.01E+03	169	this study
	ACCTGGAGCTC ATGACGGTA					7.01E+06		
OTU 4 *Desulfobulbus*	ACATCTGACTGA CCAAGCCG	57	1.000	-3.406*LOG(X) + 33.94	102.5	6.57E+03	153	this study
	TGCTGGGTGGCT AATATCCG					6.57E+06		
OTU 8 *Olivibacter*	AGTATCAACGG CACTGCTCC	57	0.998	-3.444*LOG(X) + 33.85	95.1	5.92E+03	178	this study
	ACGTGTGGGTGT TTGAAGGT					5.92E+06		
OTU 7 *Desulfobulbus*	AATTCCACATTC CCCTCTGGT	57	0.997	-2.978*LOG(X) + 33.76	116.3	4.83E+03	223	this study
	GAGGCGGCTAA TATCTGTCTCGT					4.83E+06		
OTU 5 *Azoarcus*	TCTGTCGCACTC TAGCCTTG	57	1.000	-3.247*LOG(X) + 36.58	103.2	5.31E+03	225	this study
	GGACGGGAAGA AAACTGCCA					5.31E+06		
OTU 9 *Ruminobacter*	GTTTACGGCGTG GACTACCA	57	0.999	-3.198*LOG(X) + 35.25	105.9	6.65E+03	188	this study
	ACTGCATTTGAA ACTGACGAACT					6.65E+06		
OTU 6 *Pontibacter*	GTTTACGGCGTG GACTACCA	57	1.000	-3.496*LOG(X) + 35.95	93.9	5.83E+03	183	this study
	CGTTGAAACTGC GGGTCTTG					5.83E+06		
OTU 11 *Desulfovibrio*	CGCAGTTTCAAG GGCAGTTC	57	0.999	-3.501*LOG(X) + 37.79	93.0	5.75E+03	195	this study
	GTTGCACTGTGC CAATCAGC					5.75E+06		

[1] AT = Annealing temperature in °C.

Table S2B. MIQE guidelines checklist for evaluation of qPCR primers.

Item	Importance	Status	Remarks
**Experimental design**			
definition of experimental and control groups	E	OK	Baseline, SARA 1, Challenge break, SARA 2
number within each group	E	OK	two biological replicates per sample, two technical replicates per pooled biological replicates
assay carried out by core lab or investigator's lab?	D	OK	Investigator's lab
acknowledgement of authors' contributions	D	OK	done
**Sample**			
description	E	OK	DNA isolated from rumen papillae biopsies
volume/mass of sample processed	D	OK	2 x 250 mg rumen papillae
microdissection or macrodissection	E	OK	macrodissection
processing procedure	E	OK	DNA isolation with PowerSoil DNA isolation kit
if frozen - how and how quickly?	E	OK	within 15 minutes frozen at -80°C
if fixed - with what, how quickly?	E	OK	within 5 minutes fixed in RNAlater
sample storage conditions and duration	E	OK	1 - 6 months stored at -80°C, after thawing samples were processed within 5 minutes
**Nucleic acid extraction**			
procedure and/or instrumentation	E	OK	PowerSoil DNA isolation kit, with mechanical lysis
name of kit and details of any modifications	E	OK	PowerSoil DNA Isolation Kit (MO BIO Laboratories, Inc., California, USA)
source of additional reagents used	D	OK	DNA eluted in DEPC-treated water
details of DNase or RNase treatment	E	OK	no treatment
contamination assessment (DNA or RNA)	E	OK	NTC of DNA isolation kit was analyzed using the same sequencing technology as used for samples
nucleic acid quantification	E	OK	Qubit 2.0 Fluorimeter (Qubit dsDNA BR Assay Kit, Thermo Fisher Scientific, Vienna, Austria)
instrument and method	E	OK	Qubit 2.0 Fluorimeter (Qubit dsDNA BR Assay Kit, Thermo Fisher Scientific, Vienna, Austria)
purity (a260/a280)	D		
yield	D	OK	10-150ng/µl
RNA integrity method/instrument	E	OK	not relevant
RIN/RQI or Cq of 3' and 5' transcripts	E	OK	not relevant

electrophoresis traces	D	OK	not relevant
inhibition testing (Cq dilutions, spike or other)	E	OK	not necessary, no inhibition was observed
**qPCR target information**			
if multiplex, efficiency and LOD of each assay.	E	OK	no multiplexing
sequence accession number	E		
location of amplicon	D	OK	16SrRNA V345
amplicon length	E	OK	see Table S2
in silico specificity screen (blast, etc)	E	OK	Primer3, NCBI primer designing tool, TestPrime arb-SILVA
pseudogenes, retropseudogenes or other homologs?	D		not relevant
sequence alignment	D		not relevant
secondary structure analysis of amplicon	D	OK	done
location of each primer by exon or intron (if applicable)	E		not relevant
what splice variants are targeted?	E		not relevant
**qPCR oligonucleotides**			
primer sequences	E	OK	see Table S2
RTPrimerdb identification number	D	OK	not relevant, as all of them are unpublished newly designed primers
probe sequences	D	OK	not relevant, as no probes were used
location and identity of any modifications	E	OK	no modifications
manufacturer of oligonucleotides	D	OK	Microsynth (Balgach Switzerland)
purification method	D	OK	desalted
**qPCR protocol**			
complete reaction conditions	E	OK	See main manuscript
reaction volume and amount of cDNA/DNA	E	OK	reaction volume = 20 µl, amount of DNA = 1 µl, 1:10 dilution, hypothetically 2 – 50 ng/µl DNA
primer, (probe), Mg++ and dNTP concentrations	E	OK	see manual Brilliant III Ultra-Fast SYBR Green qPCR Master Mix (Agilent, Vienna, Austria), primer = 5mM
polymerase identity and concentration	E	OK	see manual Brilliant III Ultra-Fast SYBR Green qPCR Master Mix (Agilent, Vienna, Austria)
buffer/kit identity and manufacturer	E	OK	Brilliant III Ultra-Fast SYBR Green qPCR Master Mix (Agilent, Vienna, Austria)
exact chemical constitution of the buffer	D	OK	see manual Brilliant III Ultra-Fast SYBR Green qPCR Master Mix (Agilent, Vienna, Austria)
additives (SYBR green I, DMSO, etc.)	E	OK	SYBR Green is included in the supermix; no further additives

manufacturer of plates/tubes and catalog number	D	OK	MicroAmp optical tube (0.2 µl; Applied Biosystems by life technologies)
complete thermocycling parameters	E	OK	95°C for 3 min and 40 cycles of 95°C for 5 s followed by 20 s at 57°C, melting curve 70°C to 90°C
reaction setup (manual/robotic)	D	OK	manual
manufacturer of qPCR instrument	E	OK	Stratagene Mx3000P real-time PCR System (Agilent Technologies, Santa Clara, USA)
**qPCR validation**			
evidence of optimisation (from gradients)	D	OK	not relevant
specificity (gel, sequence, melt, or digest)	E	OK	melting curve
for SYBR green, Cq of the NTC	E	OK	no amplification
standard curves with slope and y-intercept	E	OK	see Table S2
PCR efficiency calculated from slope	E	OK	see Table S2
confidence interval for PCR efficiency or standard error	D		
R2 of standard curve	E	OK	see Table S2
linear dynamic range	E	OK	see Table S2
Cq variation at lower limit	E	OK	not tested
confidence intervals throughout range	D		
evidence for limit of detection	E	OK	not tested
if multiplex, efficiency and LOD of each assay.	E	OK	not relevant, no multiplexing
**data analysis**			
qPCR analysis program (source, version)	E	OK	Stratagene Mx3000P real-time PCR System (Agilent Technologies, Santa Clara, USA)
Cq method determination	E	OK	Stratagene Mx3000P real-time PCR System settings (baseline subtracted curve fit, single threshold, automatically calculated). Threshold manually curated for maximum efficiency within linear range for each plate
outlier identification and disposition	E	OK	
results of NTCs	E	OK	no amplificate
justification of number and choice of reference genes	E	OK	not relevant
description of normalisation method	E	OK	not relevant
number and concordance of biological replicates	D	OK	not relevant

number and stage (RT or qPCR) of technical replicates	E	OK	2 technical replicates for all samples were analyzed by qPCR without additional replicates; standards were applied in 2 technical replicates
repeatability (intra-assay variation)	E	OK	repeatable
reproducibility (inter-assay variation, %CV)	D	OK	not determined (strongly recommended for clinical/diagnostic applications, but not other assays)
power analysis	D	OK	
statistical methods for result significance	E	OK	Oneway ANOVA with Tukey test
software (source, version)	E	OK	IBM SPSS (version 22, IBM Vienna, Austria)
Cq or raw data submission using RDML	D		

E = essential, D = recommended

6
