# Supplementary material for: Epimural Indicator Phylotypes of Transiently-Induced Subacute Ruminal Acidosis in Dairy Cattle
Source: Front Microbiol. 2016 Mar 4;7:274. doi: 10.3389/fmicb.2016.00274 (PMC4777738; doi:10.3389/fmicb.2016.00274)
Supplement: Supplementary file 3 [file Table3.PDF]

Table S3. Mean rumen pH, rumen pH below 5.8 in minutes per day, minimum rumen pH and maximum pH at the day before the sampling time points B (baseline), S1 (SARA 1), CB (challenge break) and S2 (SARA 2).

	Sampling time point			
	B	S1	CB	S2
**Cow 1**				
Mean pH	6.42	6.16	6.54	5.88
pH below 5.8 in min/d	0	0	0	550
Minimum pH	6.15	5.85	6.30	5.33
Maximum pH	6.62	6.54	6.75	6.39
**Cow 2**				
Mean pH	6.11	5.72	5.96	5.88
pH below 5.8 in min/d	0	850	240	430
Minimum pH	5.83	4.96	5.59	5.57
Maximum pH	6.37	6.20	6.25	6.22
**Cow 3**				
Mean pH	6.56	5.94	6.52	6.07
pH below 5.8 in min/d	0	320	0	310
Minimum pH	6.24	5.59	6.29	5.35
Maximum pH	6.96	6.27	6.73	6.54
**Cow 4**				
Mean pH	6.26	5.90	6.31	5.60
pH below 5.8 in min/d	0	330	0	1030
Minimum pH	6.00	4.87	6.11	5.13
Maximum pH	6.52	6.32	6.52	6.18
**Cow 5**				
Mean pH	6.45	6.34	6.27	5.73
pH below 5.8 in min	0	0	0	820
Minimum pH	6.30	6.10	5.96	5.21
Maximum pH	6.60	6.53	6.47	6.22
**Cow 6**				
Mean pH	6.49	6.08	6.31	5.65
pH below 5.8 in min	0	110	0	910
Minimum pH	6.19	5.42	6.07	5.02
Maximum pH	6.75	6.51	6.57	6.26
**Cow 7**				
Mean pH	6.55	6.30	6.40	5.88
pH below 5.8 in min/d	0	10	0	450
Minimum pH	6.31	5.79	6.22	5.43
Maximum pH	6.78	6.73	6.60	6.25
**Cow 8**				
Mean pH	6.35	5.86	6.31	5.56
pH below 5.8 in min/d	0	220	0	660
Minimum pH	6.01	5.13	6.10	4.75
Maximum pH	6.63	6.40	6.54	6.09
