# Supplementary material for: Epimural Indicator Phylotypes of Transiently-Induced Subacute Ruminal Acidosis in Dairy Cattle
Source: Front Microbiol. 2016 Mar 4;7:274. doi: 10.3389/fmicb.2016.00274 (PMC4777738; doi:10.3389/fmicb.2016.00274)
Supplement: Supplementary file 4 [file Table4.pdf]

Table S4. Table showing the 50 most abundant OTUs, the number of sequences, their relative abundance and their respective best NCBI BLASTn hit including the habitat of the best uncultured NCBI BLASTn hit.

OTU	No. of sequences	Relative abundance median [%]	Taxonomy NCBI BLAST cultured	Similarity [%]	Taxonomy NCBI BLAST uncultured	Similarity [%]	Habitat of best uncultured NCBI BLAST hit
1	175683	16.49	*Campylobacter fetus*; KF052122.1	98	Uncultured rumen bacterium; LN613062.1	97	goat rumen mucosa
2	75116	7.05	*Kingella oralis*; KM225757.1	95	Uncultured rumen bacterium clone; HQ400365.1	99	rumen tissue from HEAN (Hereford x Angus) crossbred beef cattle
3	48922	4.59	*Brachymonas denitrificans*; EU434449.1	96	Uncultured rumen bacterium clone; LN613041.1	99	goat rumen mucosa
4	30179	2.83	*Desulfobulbus* sp. oral taxon; GU398208.1	96	Uncultured rumen bacterium clone; GU304430.1	99	rumen epithelium Bos taurus Hereford x Angus hybrid (HEAN)
5	17000	1.60	*Azoarcus* sp.; AP012304.1	94	Uncultured bacterium; AB456263.1	95	activated sludge
6	13579	1.27	*Bacteroidetes* bacterium; AY548787.1	90	Uncultured *Bacteroidetes/Chlorobi* group bacterium clone; EU266837.1	93	tar-oil contaminated aquifer sediments
7	17416	1.63	*Desulfobulbus* sp. oral taxon; GU398207.1	97	Uncultured bacterium clone; EU844167.1	99	bovine rumen fluid fiber adherent microbiome from steer 64; 6 week sample; 1 hour after feeding
8	17786	1.67	*Bacteroidia* bacterium feline oral taxon; KM462114.1	90	Uncultured bacterium clone; EU773657.1	99	Capybara feces
9	17000	1.60	*Ruminobacter* sp.; AB730720.1	96	Uncultured rumen bacterium; AB615192.1	100	cattle rumen
10	9875	0.93	*Clostridium oceanicum*; LC007108.1	90	Uncultured rumen bacterium; LN612980.1	99	goat rumen mucosa
11	11045	1.04	*Desulfovibrio* sp. feline oral taxon; KM461955.1	97	Uncultured bacterium; HE681397.1	97	human subgingival plaque
12	10107	0.95	*Desulfobulbus* sp. oral taxon; GU398208.1	96	Uncultured rumen bacterium; LN612946.1	98	goat rumen mucosa
13	9159	0.86	Methanogenic prokaryote enrichment culture; KC821455.1	91	Uncultured rumen bacterium clone; GU303307.1	99	rumen epithelium Bos taurus Hereford x Angus hybrid (HEAN)
14	7634	0.72	*Cardiobacterium* sp. canine oral taxon; JN713405.1	91	uncultured rumen bacterium; AM884040.1	99	rumen epithelium of wethers

15	5870	0.55	*Bacteroidetes* bacterium; AY548787.1	90	Uncultured bacterium isolate; HQ222748.1	93	hybrid wastewater stabilization pond treating domestic wastewater
16	8021	0.75	*Bacteroidia* bacterium feline oral taxon; KM462114.1	91	Uncultured rumen bacterium clone; EU719248.1	98	rumen Bos taurus breed Holstein
17	7903	0.74	*Succiniclasticum ruminis*; NR_026205.1	96	Uncultured rumen bacterium; AB612616.1	99	cattle rumen
18	8838	0.83	*Gilliamella apicola*; KF600145.1	85	Uncultured rumen bacterium clone; GU303441.1	99	rumen epithelium Bos taurus Hereford x Angus hybrid (HEAN)
19	7936	0.74	*Enterobacteriaceae* bacterium; KF600324.1	86	Uncultured bacterium; AB506532.1	100	sheep rumen
20	5027	0.47	GN02 bacterium canine oral taxon; JN713535.1	93	Uncultured rumen bacterium; LN612908.1	99	goat rumen mucosa
21	6172	0.58	*Bacteroidia* bacterium feline oral taxon; KM462114.1	90	Uncultured rumen bacterium clone; GQ326919.1	98	rumen of cows
22	6439	0.60	*Synergistales* bacterium canine oral taxon; JN713408.1	96	uncultured rumen bacterium clone; GU304562.1	99	rumen epithelium Bos taurus Hereford x Angus hybrid (HEAN)
23	6774	0.64	*Ruminobacter* sp.; AB730720.1	98	Uncultured rumen bacterium; AB555288.1	98	cattle rumen
24	4532	0.43	*Endomicrobium proavitum*; CP009498.1	93	Uncultured rumen bacterium; AB614890.1	99	cattle rumen
25	5950	0.56	Rumen bacterium; GU324404.1	99	Uncultured rumen bacterium; LN612775.1	99	goat rumen mucosa
26	4926	0.46	*Desulfovibrio* sp. oral taxon; GU398181.1	96	Uncultured organism clone; HQ787847.1	96	gastrointestinal specimens Homo sapiens
27	5562	0.52	*Ruminobacter* sp.; AB730720.1	98	Uncultured rumen bacterium; AB614659.1	99	cattle rumen
28	3807	0.36	*Clostridium oceanicum*; LC007108.1	91	uncultured rumen bacterium clone; GQ327200.1	99	rumen of cows
29	3996	0.38	*Pyramidobacter piscolens*; NR_113185.1	94	uncultured rumen bacterium clone; JF797534.1	99	rumen dairy cattle (Holstein)
30	4884	0.46	*Bacteroidetes* bacterium oral taxon; GU413618.1	88	Uncultured bacterium; AB507597.1	98	sheep rumen
31	2280	0.21	*Saccharofermentans* sp.; AB730782.1	99	Uncultured bacterium clone; EU844212.1	99	rumen fluid fiber adherent microbiome from steer 64; 6 week sample; 1 hour after feeding

32	4225	0.40	*Bacteroidales bacterium*; AB730712.1	87	Uncultured rumen bacterium; AB555059.1	99	cattle rumen
33	3723	0.35	*Endomicrobium proavitum*; CP009498.1	94	Uncultured rumen bacterium; AB612798.1	99	cattle rumen
34	2634	0.25	*Bacteroidetes* bacterium; AB849455.1	97	uncultured rumen bacterium clone; GQ327735.1	99	rumen of cows
35	2911	0.27	*Clostridium oceanicum*; LC007108.1	91	Uncultured rumen bacterium clone; GQ327200.1	99	rumen of cows
36	3307	0.31	*Clostridiales* bacterium; AB730671.1	94	Uncultured rumen bacterium clone; GU303900.1	99	rumen contents Bos taurus Hereford x Angus hybrid (HEAN)
37	3650	0.34	*Fretibacterium* sp. feline oral taxon; KM462182.1	95	Uncultured rumen bacterium clone; GU304528.1	99	rumen epithelium Bos taurus Hereford x Angus hybrid (HEAN)
38	3137	0.29	*Christensenella minuta*; NR_112900.1	90	Uncultured rumen bacterium clone; GU303307.1	99	rumen epithelium Bos taurus Hereford x Angus hybrid (HEAN)
39	3113	0.29	*Deltaproteobacterium*; AY771935.1	95	Uncultured bacterium clone; JX097311.1	95	German Wadden Sea tidal flat sediments
40	3028	0.28	*Bacteroidia* bacterium feline oral taxon; KM462114.1	91	Uncultured rumen bacterium; AB616493.1	95	cattle rumen
41	2877	0.27	*Elusimicrobium minutum*; NR_074114.1	96	Uncultured *Termite* group 1 bacterium; AM491075.1	99	Cow Rumen
42	2259	0.21	*Clostridiales* bacterium canine oral taxon; JN713385.1	94	Uncultured rumen bacterium; AB185640.1	99	PCR-derived sequence from cattle rumen
43	3561	0.33	*Clostridiales* bacterium; AB730671.1	94	uncultured rumen bacterium clone; GU303900.1	99	sheep rumen
44	3302	0.31	*Succinivibrio dextrinosolvens*; AB849337.1	99	Uncultured *Succinivibrio* sp. clone; KM073420.1	99	rumen Budorcas taxicolor bedfordi
45	3434	0.32	*Succinivibrio dextrinosolvens*; AB849337.1	96	uncultured bacterium; AB821776.1	99	cattle rumen
46	2827	0.27	*Bacteroidia* bacterium feline oral taxon; KM462114.1	90	uncultured bacterium; AB746546.1	96	cattle rumen
47	2867	0.27	*Clostridiales* bacterium feline oral taxon; KM462113.1	95	uncultured bacterium; KM073392.1	98	rumen Budorcas taxicolor bedfordi
48	2934	0.28	*Clostridium* from anoxic bulk soil; AJ229251.1	100	uncultured rumen bacterium clone; JX218666.1	99	cattle rumen

49	3141	0.29	*Selenomonas ruminantium* subsp. *lactilytica*; NR_075026.1	99	uncultured rumen bacterium clone; AB555463.1	99	cattle rumen
50	3132	0.29	*Bacteroidia* bacterium feline oral taxon; KM462114.1	91	Uncultured rumen bacterium; AB612732.1	96	cattle rumen
