# Supplementary material for: Epimural Indicator Phylotypes of Transiently-Induced Subacute Ruminal Acidosis in Dairy Cattle
Source: Front Microbiol. 2016 Mar 4;7:274. doi: 10.3389/fmicb.2016.00274 (PMC4777738; doi:10.3389/fmicb.2016.00274)
Supplement: Supplementary file 5 [file Table5.PDF]

Table S5. Relative abundances of 50 most abundant genera.

Genus	Relative abundance [%]
Campylobacter	16.31
Kingella	7.16
Desulfobulbus	5.08
Brachymonas	4.97
Pseudosphingobacterium	4.43
Alkalibaculum	3.59
Ruminococcus	3.41
Acidaminobacter	3.28
Anaerophaga	2.87
Oscillibacter	2.84
Lutispora	2.56
Saccharofermentans	2.31
Ruminobacter	2.21
Succinivibrio	1.74
Flavonifractor	1.68
Azospira	1.63
Acetivibrio	1.60
Olivibacter	1.42
Desulfovibrio	1.42
Elusimicrobium	1.26
Fastidiosipila	1.22
Rikenella	1.16
Succiniclasticum	1.04
Anaerovibrio	1.02
Ornithobacterium	0.93
Sporobacter	0.91
Papillibacter	0.85
Coprobacillus	0.78
Dongia	0.75
Suttonella	0.72
Thioreductor	0.56
Aminobacterium	0.53
Clostridium_IV	0.51
Pseudoflavonifractor	0.51
Anaerorhabdus	0.50
Tannerella	0.48
Cellulosilyticum	0.45
Dehalobacter	0.45
Selenomonas	0.45
Gracilibacter	0.44
Clostridium_XlVb	0.42
Pyramidobacter	0.41
Brevinema	0.41

Syntrophococcus	0.41
Holdemania	0.40
Thermovirga	0.33
Bifidobacterium	0.33
Butyrivibrio	0.32
Bilophila	0.32
Meniscus	0.31
