# Supplementary material for: Epimural Indicator Phylotypes of Transiently-Induced Subacute Ruminal Acidosis in Dairy Cattle
Source: Front Microbiol. 2016 Mar 4;7:274. doi: 10.3389/fmicb.2016.00274 (PMC4777738; doi:10.3389/fmicb.2016.00274)
Supplement: Supplementary file 6 [file Table6.PDF]

Table S6. Phyla statistics. Phyla with significant changes in relative abundance over the feeding experiment are depicted. B = baseline, S1 = SARA challenge 1, CB = challenge break and S2 = SARA challenge 2.

	**Median relative abundance [%]**					
	**Sampling time point**					
**Phylum**	**B**	**S1**	**CB**	**S2**	**SEM**	***P*-value**
Firmicutes	32.52^{b}	40.89	53.62^{aA}	40.65^{B}	1.71	0.001
Proteobacteria	48.30^{a}	41.55^{b}	30.07^{b}	40.26	1.70	0.005
Spirochaetes	1.00^{a}	0.29^{b}	0.33^{b}	0.21^{b}	0.07	<0.001
Lentisphaerae	0.22^{a}	0.02^{b}	0.03^{b}	0.02^{b}	0.03	0.001
Tenericutes	0.07^{a}	0.05	0.07^{a}	0.02^{b}	0.01	0.027
Deferribacteres	0.02^{aA}	0.01^{B}	0.00^{b}	0.00^{b}	<0.01	0.002
Deinococcus-Thermus	0.01^{a}	0.00^{b}	0.00^{b}	0.00^{b}	<0.01	<0.001

[1] For significance, *P*-values ≤ 0.05 and for a trend 0.05 < *P* ≤ 0.10 were chosen.
