# Supplementary material for: Epimural Indicator Phylotypes of Transiently-Induced Subacute Ruminal Acidosis in Dairy Cattle
Source: Front Microbiol. 2016 Mar 4;7:274. doi: 10.3389/fmicb.2016.00274 (PMC4777738; doi:10.3389/fmicb.2016.00274)
Supplement: Supplementary file 7 [file Table7.PDF]

Table S7. OTUs with significant shifts in relative abundance at four sampling time points B (baseline), S1 (SARA 1), CB (challenge break) and S2 (SARA 2) are depicted. Only statistically significant shifts or trends from 50 most abundant OTUs are shown.

		Median relative abundance [%]					
		Sampling time point					
OTU	Taxonomy	B	S1	CB	S2	SEM	*P*-value
1	*Campylobacter*	19.83^{A}	18.84	9.26^{B}	15.15	1.29	0.074
2	*Kingella*	10.85^{a}	3.43^{b}	3.13^{b}	6.84^{b}	0.91	0.001
5	*Azoarcus*	2.17^{a}	0.89^{b}	0.57^{b}	0.86^{b}	0.26	0.005
9	*Ruminobacter*	0.04^{b}	0.29^{b}	3.02^{a}	0.03^{b}	0.51	0.003
18	*Vibrio*	0.00^{b}	0.37^{b}	1.47^{a}	0.01^{b}	0.16	≤0.001
20	*Altererythrobacter*	1.39^{a}	0.06^{b}	0.21^{b}	0.05^{b}	0.12	≤0.001
22	*Aminobacterium*	0.47	0.48^{b}	0.29^{B}	0.88^{aA}	0.07	0.032
23	*Ruminobacter*	0.02^{b}	0.09^{b}	1.10^{a}	0.08^{b}	0.13	0.001
26	*Desulfovibrio*	0.36	0.25^{B}	0.35	0.82^{A}	0.06	0.054
30	*Alistipes*	0.00^{b}	0.6	0.06^{b}	1.16^{a}	0.11	0.003
34	*Eubacterium*	0.16^{B}	0.24	0.47^{Aa}	0.06^{b}	0.42	0.007
37	*Aminobacterium*	0.14^{b}	0.32	0.16^{b}	0.58^{a}	0.05	0.013
42	*Desulfotomaculum*	0.1	0.31	0.28^{A}	0.08^{B}	0.03	0.054
47	*Desulfotomaculum*	0.10^{B}	0.23	0.2	0.32^{A}	0.03	0.062
49	*Selenomonas*	0.01^{b}	0.14^{B}	0.58^{aA}	0.15^{b}	0.08	0.007

Significant differences between sampling time points ($P \leq 0.05$) in rows are marked with different letters as un-capitalized- and trends ($P \leq 0.10$) with different capitalized upper-case superscripts.
