# Supplementary material for: Epimural Indicator Phylotypes of Transiently-Induced Subacute Ruminal Acidosis in Dairy Cattle
Source: Front Microbiol. 2016 Mar 4;7:274. doi: 10.3389/fmicb.2016.00274 (PMC4777738; doi:10.3389/fmicb.2016.00274)
Supplement: Supplementary file 8 [file Table8.PDF]

Table S8. Comparison of correlation analysis of OTUs (and all bacteria) with average ruminal pH calculated with qPCR data and sequencing data. Only OTUs with available qPCR data are shown.

Factor correlated with average ruminal pH	qPCR		Illumina sequencing	
	Correlation	*P*-value	Correlation	*P*-value
All bacteria	-0.15	0.421	NA	NA
OTU 1	-0.31	0.088	-0.01	0.961
OTU 2	0.05	0.769	0.26	0.144
OTU 5	0.03	0.867	0.28	0.119
OTU 9	0.18	0.328	0.27	0.143

NA = not available
