# Supplementary material for: Epimural Indicator Phylotypes of Transiently-Induced Subacute Ruminal Acidosis in Dairy Cattle
Source: Front Microbiol. 2016 Mar 4;7:274. doi: 10.3389/fmicb.2016.00274 (PMC4777738; doi:10.3389/fmicb.2016.00274)
Supplement: Supplementary file 9 [file Image1.PDF]

Figure S1.

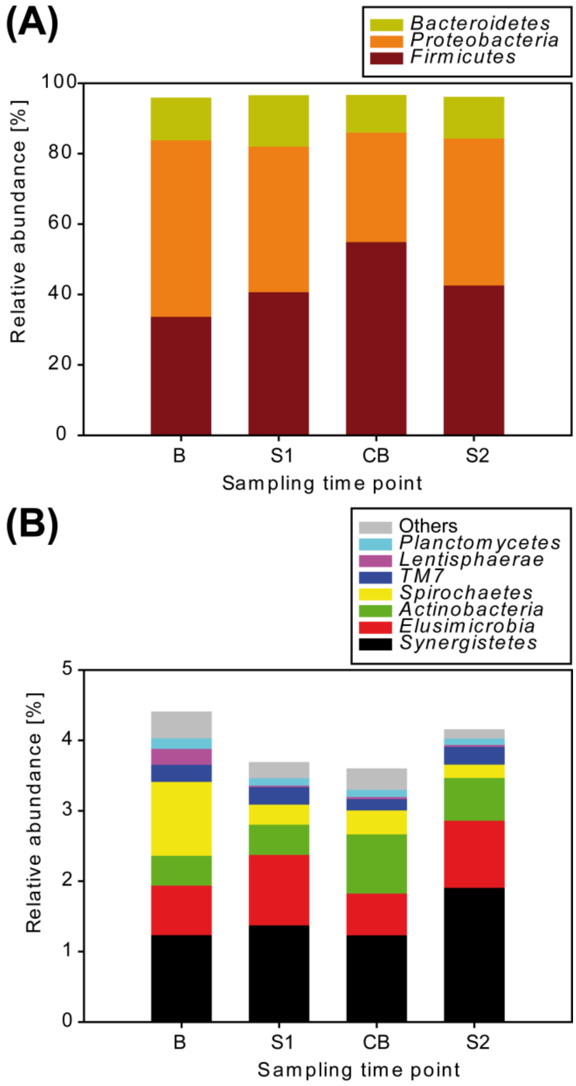

Figure S1. Relative abundance of phyla detected in four sampling time points. (A) Relative abundance of three most abundant phyla. (B) Relative abundance of low abundant phyla. Sampling time points are as follows: B (baseline), S1 (SARA 1), CB (challenge break) and S2 (SARA 2).
