# Supplementary material for: Epimural Indicator Phylotypes of Transiently-Induced Subacute Ruminal Acidosis in Dairy Cattle
Source: Front Microbiol. 2016 Mar 4;7:274. doi: 10.3389/fmicb.2016.00274 (PMC4777738; doi:10.3389/fmicb.2016.00274)
Supplement: Supplementary file 10 [file Image2.PDF]

Figure S2.

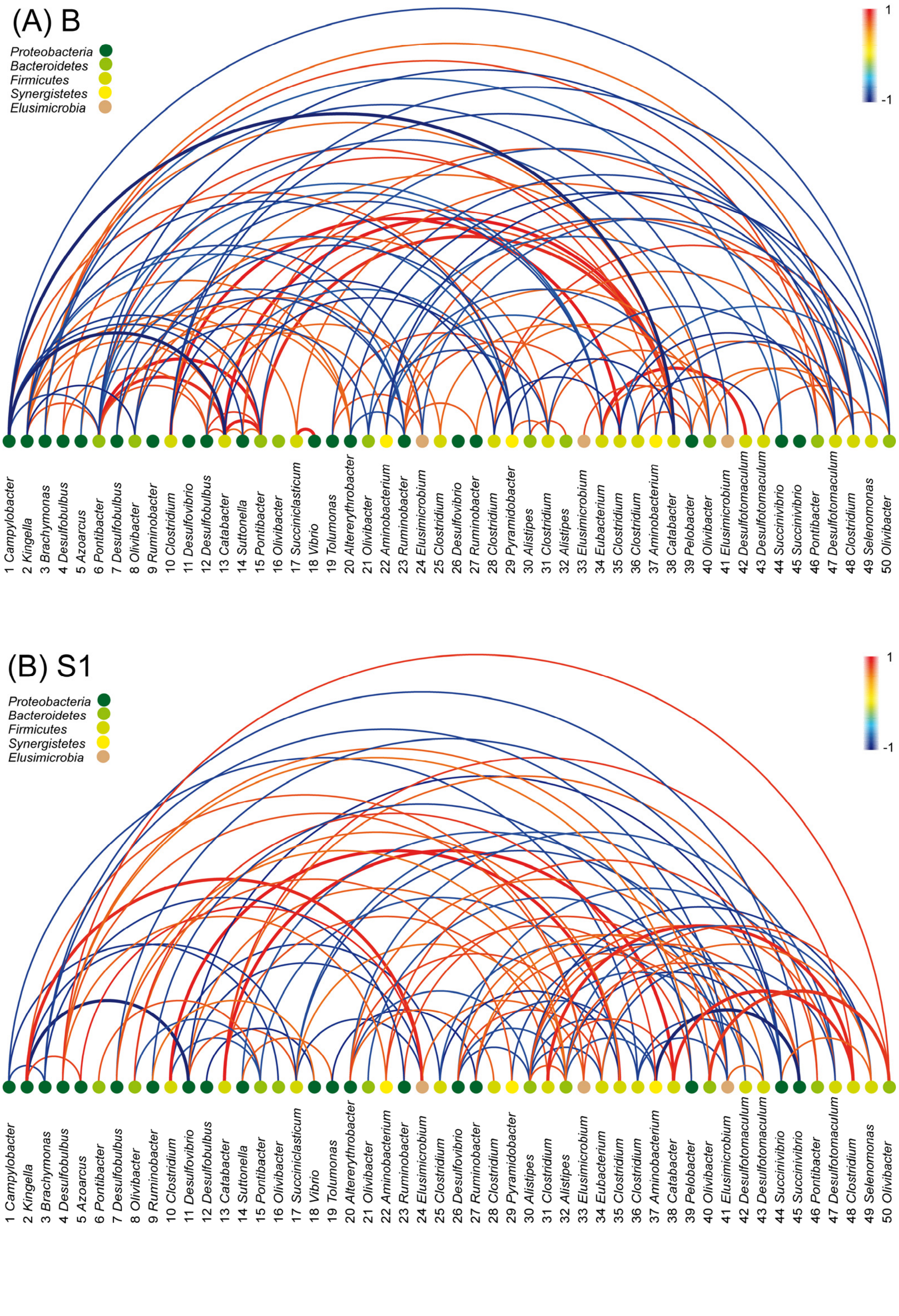
(A) B
Proteobacteria
Bacteroidetes
Firmicutes
Synergistetes
Elusimicrobia
1
-1
1 Campylobacter
2 Kingella
3 Brachymonas
4 Desulfobulbus
5 Azoarcus
6 Pontibacter
7 Desulfobulbus
8 Olivibacter
9 Ruminobacter
10 Clostridium
11 Desulfovibrio
12 Desulfobulbus
13 Catabacter
14 Suttonella
15 Pontibacter
16 Olivibacter
17 Succiniclasticum
18 Vibrio
19 Tolumonas
20 Altererythrobacter
21 Olivibacter
22 Aminobacterium
23 Ruminobacter
24 Elusimicrobium
25 Clostridium
26 Desulfovibrio
27 Ruminobacter
28 Clostridium
29 Pyramidobacter
30 Alistipes
31 Clostridium
32 Alistipes
33 Elusimicrobium
34 Eubacterium
35 Clostridium
36 Clostridium
37 Aminobacterium
38 Catabacter
39 Pelobacter
40 Olivibacter
41 Elusimicrobium
42 Desulfotomaculum
43 Desulfotomaculum
44 Succinivibrio
45 Succinivibrio
46 Pontibacter
47 Desulfotomaculum
48 Clostridium
49 Selenomonas
50 Olivibacter
(B) S1
Proteobacteria
Bacteroidetes
Firmicutes
Synergistetes
Elusimicrobia
1
-1
1 Campylobacter
2 Kingella
3 Brachymonas
4 Desulfobulbus
5 Azoarcus
6 Pontibacter
7 Desulfobulbus
8 Olivibacter
9 Ruminobacter
10 Clostridium
11 Desulfovibrio
12 Desulfobulbus
13 Catabacter
14 Suttonella
15 Pontibacter
16 Olivibacter
17 Succiniclasticum
18 Vibrio
19 Tolumonas
20 Altererythrobacter
21 Olivibacter
22 Aminobacterium
23 Ruminobacter
24 Elusimicrobium
25 Clostridium
26 Desulfovibrio
27 Ruminobacter
28 Clostridium
29 Pyramidobacter
30 Alistipes
31 Clostridium
32 Alistipes
33 Elusimicrobium
34 Eubacterium
35 Clostridium
36 Clostridium
37 Aminobacterium
38 Catabacter
39 Pelobacter
40 Olivibacter
41 Elusimicrobium
42 Desulfotomaculum
43 Desulfotomaculum
44 Succinivibrio
45 Succinivibrio
46 Pontibacter
47 Desulfotomaculum
48 Clostridium
49 Selenomonas
50 Olivibacter

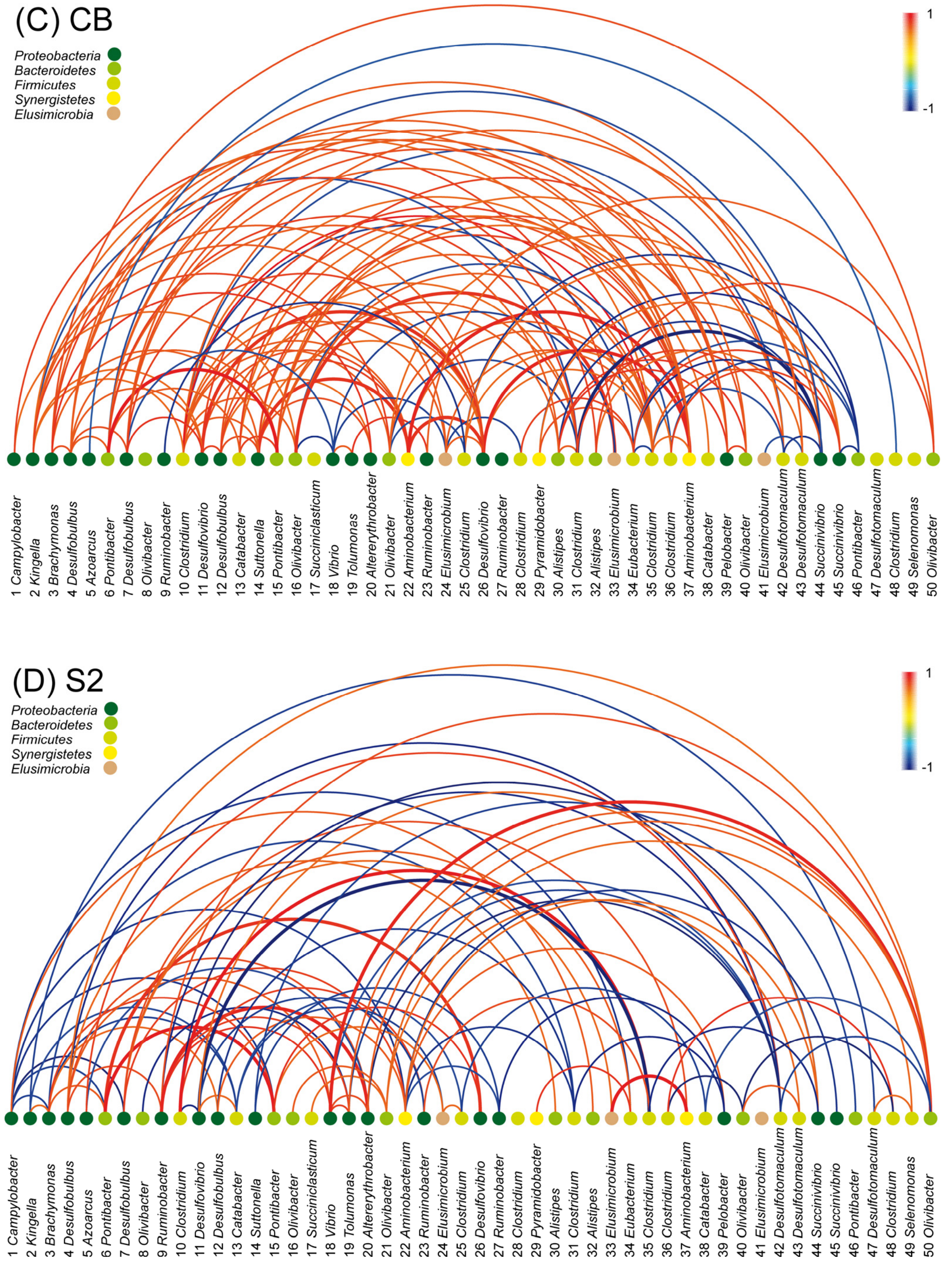

Figure S2. Arc diagrams showing correlations between the 50 most abundant OTUs at each sampling time point. Strong correlation values $|r_s|\geq 0.7$ between the OTUs (A) in sampling time point B, (B) in sampling time point S1, (C) sampling time point CB, and (D) sampling time point S2. Correlation values $|r_s|\geq 0.9$ are illustrated as bold lines. Positive correlations are shown in red and negative correlations in blue color. B = baseline, S1 = SARA challenge 1, CB = challenge break and S2 = SARA challenge 2.
